# Impact of Genetic Polymorphisms on the Metabolic Pathway of Vitamin D and Survival in Non-Small Cell Lung Cancer

**DOI:** 10.3390/nu13113783

**Published:** 2021-10-25

**Authors:** Laura Elena Pineda Lancheros, Cristina Pérez Ramírez, Almudena Sánchez Martín, José María Gálvez Navas, Fernando Martínez Martínez, María del Carmen Ramírez Tortosa, Alberto Jiménez Morales

**Affiliations:** 1 Pharmacy Service, Pharmacogenetics Unit, University Hospital Virgen de las Nieves, 18014 Granada, Spain; lepinedal@unal.edu.co (L.E.P.L.); almuweb06@gmail.com (A.S.M.); jmgalna7@gmail.com (J.M.G.N.); alberto.jimenez.morales.sspa@juntadeandalucia.es (A.J.M.); 2 Center of Biomedical Research, Department of Biochemistry and Molecular Biology II, Institute of Nutrition and Food Technology “José Mataix”, University of Granada, Avda. del Conocimiento s/n., 18016 Granada, Spain; mramirez@ugr.es; 3 Department of Pharmacy and Pharmaceutical Technology, Social and Legal Assistance Pharmacy Section, Faculty of Pharmacy, University of Granada, 18071 Granada, Spain; femartin@ugr.es

**Keywords:** vitamin D metabolism, survival, non-small-cell lung cancer, single nucleotide polymorphisms, *CYP27B1*, *CYP24A1*, *CYP2R1*, *GC*, *VDR*

## Abstract

Vitamin D has been associated with risk, development, and progression of cancer. However, the genes involved in its metabolism are highly polymorphic, compromising its activity. The aim of this study is to evaluate the association between the gene polymorphisms involved in the metabolic pathway of vitamin D and survival in patients with non-small-cell lung cancer (NSCLC). The study was designed as an observational cohort which included 194 Caucasians patients from southern Spain with NSCLC. Real-time polymerase chain reaction was used to analyze the following polymorphisms: *CYP27B1* rs4646536, rs3782130, and rs10877012; *CYP24A1* rs6068816 and rs4809957; *GC* rs7041; *CYP2R1* rs10741657; *VDR* rs1544410 (BsmI), rs11568820 (Cdx-2), rs2228570 (FokI), rs7975232 (ApaI), and rs731236 (TaqI). Progression-free survival (PFS) and overall survival were assessed. Cox regression showed that rs4646536 was associated with PFS in the general population (*p* = 0.0233) and in the non-resected NSCLC subgroup (*p* = 0.0233). In the resected NSCLC subgroup, rs11568820 was associated with OS (*p* = 0.0129) and rs7041 with PFS (*p* = 0.0447). In the non-resected NSCLC subgroup, rs6068816 was associated with PFS (*p* = 0.0048) and OS (*p* = 0.0089) and rs731236 and rs7975232 were associated with OS (*p* = 0.0005) and PFS (*p* = 0.0002), respectively. The other polymorphisms showed no effect on the results. The rs4646536, rs6068816, rs7041, rs11568820, rs731236, and rs7975232 polymorphisms are associated with survival in NSCLC and may have a substantial role as prognostic markers of the disease.

## 1. Introduction

Lung cancer is the second most commonly diagnosed type of cancer, after breast cancer, with a global incidence of around 11.4% [1]. It represents the leading cause of cancer death worldwide (18.0%) [1]. According to the latest cancer statistics, it is estimated that there will be more than 235,760 new cases and 131,880 deaths in the United States in 2021 [2].

Non-small-cell lung cancer (NSCLC) represents around 80–85% of all lung cancers [3]. Survival of lung cancer patients 5 years after diagnosis is between 10% and 20% in most countries, even after surgery, chemotherapy, and radiotherapy [4]. There is therefore a need to look for new prognostic biomarkers that will improve survival in these patients [5]. The main factor influencing disease prognosis is the initial tumor stage [6,7,8]. However, studies in patients diagnosed with the same stage have shown variability in survival, which suggests that other factors may influence the prognosis of NSCLC [9,10,11,12]. It should be emphasized that genetic alterations such as single nucleotide polymorphisms (SNPs) may be the cause of this interindividual variability in survival of patients with NSCLC [5,13,14,15].

Vitamin D is notable for its wide-ranging biological functions, which involve, among other things, suppressing metastasis by inhibiting tumor progression, angiogenesis, and cell proliferation, or by apoptosis promotion in cancer cells [5,16,17,18,19,20]. In lung cancer, specifically, in vivo and in vitro studies have been carried out, showing that 1,25-dihydroxycholecalciferol inhibits growth of lung cancerous cell lines and affects cell cycle regulation in squamous cell carcinoma models [21,22,23]. In mouse models, calcitriol proved to inhibit growth of lung tumors and metastases [24,25]. Furthermore, observational studies have found that lung cancer mortality is lower during the autumn and summer months, the times of year associated with the highest vitamin D levels [26,27]. Risk, development, and growth of solid and non-solid tumors have been associated with vitamin D low levels [19].

Vitamin D has two basic isoforms: vitamin D2 (ergocalciferol) and vitamin D3 (cholecalciferol). Both are produced endogenously following sun exposure and by direct consumption through diet or supplements [27,28,29,30]. The two isoforms of vitamin D (D2 and D3) bind to the vitamin D binding protein (VDBP), encoded by the group-specific component (vitamin D binding protein) gene (*GC*), facilitating their transport [29,31,32,33]. Both forms are subsequently metabolized in the liver to 25-hydroxycholecalciferol, by 25-hydroxylases (encoded by *CYP2R1* and *CYP27A1*), this being its main circulating form [29,31,34,35]; 1α-hydroxylase (encoded by *CYP27B1*) converts it to 1,25-dihydroxycholecalciferol, either in the kidney (where it is released into circulation) or in specific target organs, converting it to its biologically active form [16,27,28,29,31]. In the target tissues, 1,25-dihydroxycholecalciferol binds to the vitamin D receptor (VDR) and interacts with the retinoid X receptor (RXR), forming a heterodimer complex (VDR-RXR), which is translocated to the nucleus, binding to the VDR response elements in numerous genomic loci, some of which have anticancer properties [16,31,36,37]. Finally, circulating 1,25-dihydroxycholecalciferol and 25-hydroxycholecalciferol are degraded by 24-hydroxylase (encoded by *CYP24A1*) to calcitroic acid and other hydrosoluble products which are inactive and are excreted in bile or urine [27,29,31,36].

The genes that encode the enzymes involved in the vitamin D pathway are highly polymorphic [27]. These genetic alterations may influence the expression of those genes in lung tumor tissue, modifying the activity of vitamin D [15,28,36,38,39,40,41]. Therefore, they may play a vital part in the development, progression, and prognosis of NSCLC [5]. In this context, polymorphisms in the genes that mediate the metabolic pathway of vitamin D (*CYP27B1*, *CYP24A1*, *CYP2R1*, *GC*, and *VDR*) may have a crucial role in the survival of patients with NSCLC [5,14,15,31,42,43].

On the basis of the foregoing, this study was design to evaluate the association of SNP-type polymorphisms in the genes implicated in the vitamin D metabolic pathway with progression-free survival (PFS) and overall survival (OS) in Caucasian patients (from Spain) with NSCLC.

## 2. Materials and Methods

### 2.1. Study Design

A prospective observational cohort study was carried out.

### 2.2. Ethics Statement

This study was conducted with the approvement of the Ethics and Research Committee of the Sistema Andaluz de Salud (Andalusian Health Service) (SAS) and in accordance with the Declaration of Helsinki (code: 1322-N-20). A written informed consent form was signed by the patients for collection of saliva or blood samples and its further donation to the biobank. The confidentiality of the sample treatment was ensured through their codification.

### 2.3. Study Population

The study included 194 patients of Caucasian origin from southern Spain with NSCLC, recruited in the Hospital Universitario Virgen de las Nieves, Granada, Spain, diagnosed between 2003 and 2019 and followed up until December 2020. The inclusion criteria for the patient group were age 18 years or over, confirmed histologic or cytologic diagnosis of NSCLC (stages I-IV), adequate organ function, measurable disease on computed tomography, with no previous treatment and available clinical data. The patients were treated in accordance with the National Comprehensive Cancer Network (NCCN) guidelines [44].

### 2.4. Sociodemographic and Clinical Variables

From the clinical records we collected sociodemographic information, including family history of cancer, gender, smoking status, previous lung disease, drinking status, body mass index (BMI), and age at diagnosis. Individuals were classified as active smokers if they had smoked 100 or more cigarettes in their lives and currently smoked, as ex-smokers if they had smoked 100 or more cigarettes in their lives but did not currently smoke, and as non-smokers if they had never smoked or had smoked fewer than 100 cigarettes in their lives. Individuals were classified by standard drink units (SDUs) as non-drinkers if they were teetotalers or did not consume alcohol regularly, as active drinkers if their alcohol consumption was greater than 4 SDUs per day in men and greater than 2.5 SDUs per day in women, and as ex-drinkers if their alcohol consumption was greater than 4 SDUs per day in men and greater than 2.5 SDUs per day in women, but they did not currently drink [45]. Histopathologic data (tumor histology and stage) and first-line treatment were also collected. The guidelines of the AJCC staging system criteria were followed in the tumor classification [46].

### 2.5. Genetic Variables

#### 2.5.1. DNA Isolation

The DNA samples, isolated from saliva or blood, were obtained from the Biobank of the Hospital Universitario Virgen de las Nieves, which is part of the SAS Biobank. BD Falcon^TM^ 50 mL conical tubes were used in saliva samples collection (BD, Plymouth, UK). BD Vacutainer^®^ tubes with anticoagulant were (3 mL of EDTA K3) were used in blood samples collection. QIAamp DNA Mini kit (Qiagen GmbH, Hilden, Germany) were used in the DNA extraction performance, following the specifications provided by the manufacturer for purification of DNA from saliva or blood, and stored at −40 °C. The concentration and purity of the DNA were assessed using a NanoDrop 2000™ UV spectrophotometer with 280/260 and 280/230 absorbance ratios.

#### 2.5.2. Detection of Gene Polymorphisms

We determined the gene polymorphisms by real-time PCR allelic discrimination assay using TaqMan^®^ probes (ABI Applied Biosystems, QuantStudio 3 Real-Time PCR System), following the manufacturer’s instructions (Table 1).

### 2.6. Survival Variables

PFS and OS were used in survival measurement.

Survival was measured by PFS and OS. We evaluated OS as the time from cancer diagnosis to death or final follow-up and calculated PFS as the time from start of treatment to last known follow-up, death, or relapse. The mortality data were obtained from the clinical histories and the Granada population-based cancer registry.

### 2.7. Statistical Analysis

The quantitative data were expressed as the mean (plus/minus standard deviation) for variables with normal distribution or medians and percentiles (25 and 75) for variables with non-normal distribution. Normality was assessed with the usage of the Shapiro–Wilk test.

We used the Kaplan–Meier method and log-rank test to analyze the associations between survival and the demographic, genetic, and clinical variables. The Cox proportional hazards regression model was used for the multivariate analysis (stepwise backward selection method) to obtain the adjusted hazard ratio (HR) and the 95% confidence interval (95% CI) for the possible survival prognostic factors.

All the tests were bilateral with a significance level of *p* < 0.05. R 4.0.2 software was used to perform the data analysis [47].

The linkage disequilibrium, Hardy–Weinberg equilibrium, and haplotype frequency were determined through the D’ and r2 coefficients and were estimated using the PLINK and Haploview 4.2 programs [48,49].

## 3. Results

### 3.1. Patient Characteristics

A total of 194 Caucasian patients from southern Spain with NSCLC were included in the study. Their sociodemographic, clinical, and pathologic characteristics are summarized in Table 2. The mean age of the patients was 60.86 ± 10.51 years, 141 were men (141/194; 72.68%), and 130 were stage IIIB-IV (130/194; 67.36%). Surgery was the first course of treatment for 48 patients (48/194; 24.74%), of whom 97.91% (47/48) had stage I-IIIA. During follow-up, 154 fatal events were recorded. For all patients, the median OS and PFS were 26.8 (15.5–64.20 and 13.7 (6.46–29.9) months, respectively.

### 3.2. Influence of Clinical-Pathologic Characteristics on Survival

#### 3.2.1. Overall Population

Median OS was greater in women (P_log-rank_ = 0.030; 38.9 vs. 26.1 months; Table S1; Figure S1), non-drinkers (P_log-rank_ = 0.0005; 36.53 months vs. 21.43 for drinkers and 8.85 for ex-drinkers; Table S1; Figure S2), stages I, II, and IIIA (P_log-rank_ < 0.001; 107.6 vs.21.1 months; Table S1; Figure S3), and with surgery as the first course of treatment (P_log-rank_ < 0.001; 130.0 vs. 23.2 months; Table S1; Figure S4).

Median PFS was associated with previous lung disease (P_log-rank_ = 0.040; 17.6 vs. 12.3 months; Table S2; Figure S5), non-drinkers (P_log-rank_ = 0.020; 17.07 months vs. 9.17 for drinkers and 7.82 for ex-drinkers; Table S2; Figure S6), stages I, II, and IIIA (P_log-rank_ < 0.001; 29.4 vs. 10.6 months; Table S2; Figure S7), and with surgery as the first course of treatment (P_log-rank_ < 0.001; 59.0 vs. 10.9 months; Table S2; Figure S8).

#### 3.2.2. Subgroup Analysis

In the subgroup of patients with resected NSCLC, OS was associated with family history of cancer (P_log-rank_ = 0.030; 103 vs. 176 months; Table S3; Figure S9) and PFS was greater in stages I, II, and IIIA (P_log-rank_ = 0.020; 64.5 vs. 6.9 months; Table S4; Figure S10). For patients with non-resected NSCLC, median OS was greater in women (P_log-rank_ = 0.007; 30.4 vs. 20.1 months; Table S5; Figure S11), non-drinkers (P_log-rank_ = 0.002; 25.4 months vs. 18.4 for drinkers and 8.95 for ex-drinkers; Table S5; Figure S12), and in stages I, II, and IIIA (P_log-rank_ = 0.004; 45.9 vs. 21.1 months; Table S5; Figure S13). On the other hand, median PFS was associated with BMI < 24 (P_log-rank_ = 0.020; 18.8 vs. 10.2 months; Table S6; Figure S14) in patients with non-resected NSCLC.

### 3.3. Genotype Distribution

**Figure 1:**
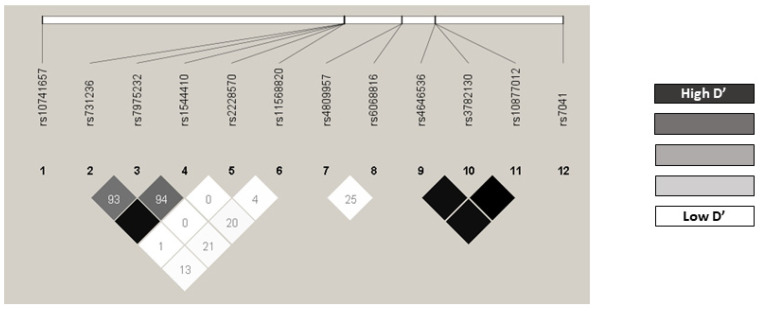
Linkage disequilibrium.

Minor allele frequencies (MAFs) greater than 1% were shown in all the polymorphisms and none of them was excluded from the analysis (Table S7). According to the Hardy–Weinberg equilibrium model, all the gene polymorphism distributions were in agreement with those expected, both for OS (Table S8) and for PFS (Table S9), with the sole exception of OS for *VDR* rs731236 (*p* = 0.0231). No statistical differences were found from the frequencies described for the Iberian population for this variant (VDR rs731236 G allele: 0.374 vs. 0.423; *p* = 0.1777) [50]. The LD’ and r2 linkage disequilibrium values are shown in Table S10. In particular, the *VDR* rs731236/rs1544410, *CYP27B1* rs4646536/rs3782130, *CYP27B1* rs4646536/rs10877012, and *CYP27B1* rs3782130/rs10877012 pairs were in strong linkage disequilibrium (Figure 1).

### 3.4. Influence of Gene Polymorphisms on Survival

#### 3.4.1. Overall Population

##### Overall Survival

**Figure 2:**
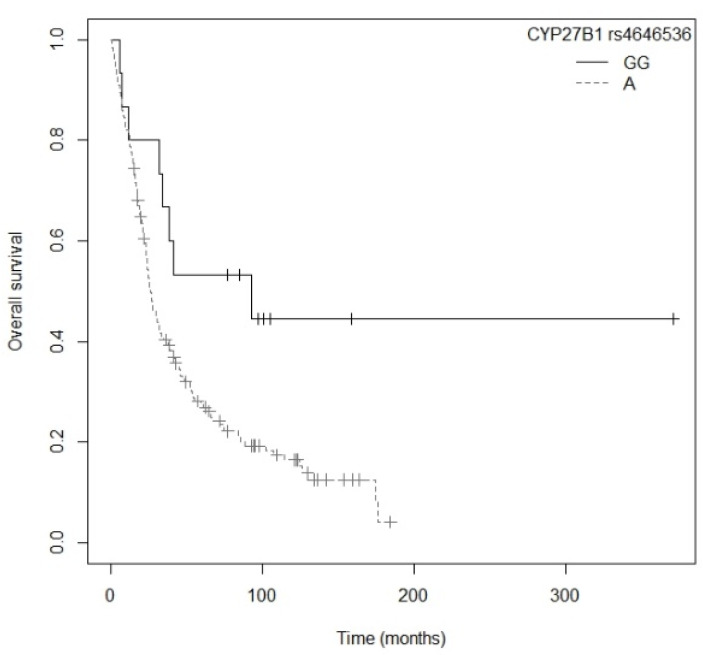
Kaplan–Meier plot of overall survival curves with the A allele of the *CYP27B1* rs4646536 gene polymorphism in 194 patients with NSCLC.

The bivariate analysis showed that OS was associated with the *CYP27B1* rs4646536 gene polymorphism (Table S11). In particular, patients carrying the A allele had a higher risk of death compared to those with the GG genotype (*p* = 0.0021; HR = 2.32; CI_95%_ = 1.13–4.73; Table S11). The Kaplan–Meier plot of OS curves with the A allele of *CYP27B1* rs4646536 is shown in Figure 2 (P_log-rank_ = 0.020). Median OS was 92.9 (CI_95%_= 34.1-not reached [NR]) months for the GG genotype. For the AG and GG genotypes, median OS was 24.9 (CI_95%_ = 22.7–38.0) and 27.0 (95% CI = 23.2–41.8) months, respectively. Cox regression adjusted for gender, tumor stage, and first-line treatment showed that the *CYP27B1* rs4646536 polymorphism exhibited a tendency toward association with OS (*p* = 0.0569; HR = 2.01; CI_95%_ = 0.98–4.14) (P_likelihood ratio test_ ≤ 2 × 10−16) (Table 3). 

##### Progression-Free Survival

**Figure 3:**
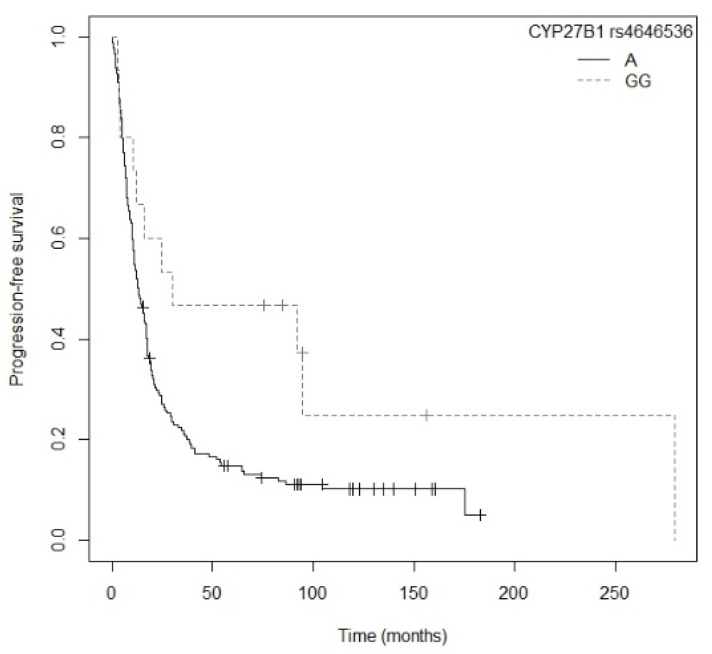
Kaplan–Meier plot of progression-free survival curves with the A allele of the *CYP27B1* rs4646536 gene polymorphism in 194 patients with NSCLC.

Patients carrying the A allele for the *CYP27B1* rs4646536 gene polymorphism showed a higher risk of progression compared to those carrying the GG genotype (*p* = 0.0266; HR = 2.07; CI_95%_ =1.08–3.93; Table S12). The Kaplan–Meier plot of PFS curves with the A allele of *CYP27B1* rs4646536 is shown in Figure 3 (P_log-rank_ = 0.020). Median PFS was 30.0 (CI_95%_ = 12.3-NR) months for the GG genotype. For AG and GG carriers, median PFS was 12.3 (CI_95%_ = 9.17–17.6) and 13.3 (CI_95%_ = 10.9–17.6) months, respectively. Cox regression adjusted for first-line treatment showed that the *CYP27B1* rs4646536 polymorphism was associated with PFS (*p* = 0.0233; HR = 2.11; CI_95%_ = 1.11–4.04) (P_likelihood ratio test_ = 1 × 10−12) (Table 4).

#### 3.4.2. Subgroup Analysis

##### Overall Survival

**Figure 4:**
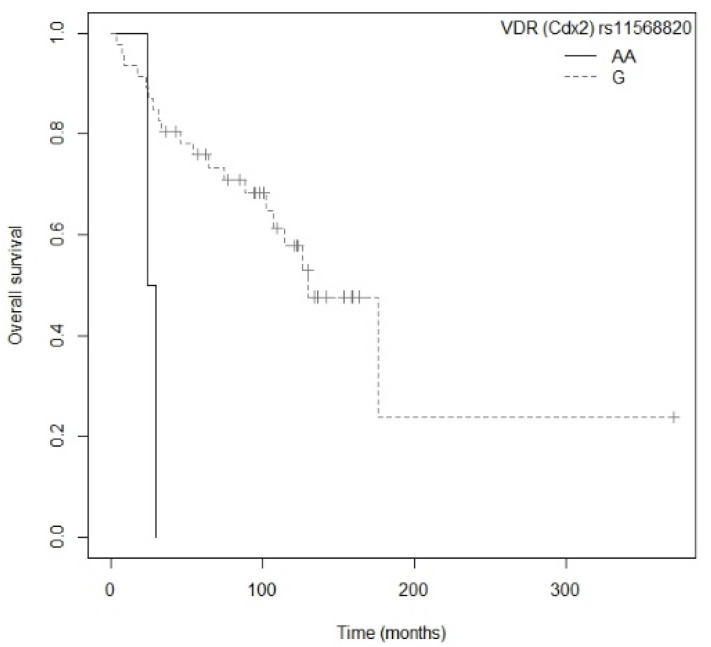
Kaplan–Meier plot of overall survival curves with the G allele of the *VDR* rs11568820 gene polymorphism in the resected NSCLC subgroup.

In the subgroup of resected NSCLC patients, those carrying the GG genotype for the *GC* rs7041 polymorphism had a higher risk of death compared to those with the T allele (*p* = 0.0242; HR = 2.72; CI_95%_ = 1.14–6.47; Table S13). Median OS for the GG genotype was 61.2 (CI_95%_ = 24.3-NR) months, while for the GT and TT genotypes it was 107.6 (CI_95%_ = 75.0-NR) and 176.1 (CI_95%_ = 126.4-NR) months, respectively. The Kaplan–Meier plot of OS curves with the T allele of *GC* rs7041 is shown in Figure S15 (P_log-rank_ = 0.020). Similarly, patients carrying the AA genotype for the *VDR* rs11568820 polymorphism were associated with a higher risk of death than those with the GG genotype (*p* = 0.0129; HR = 7.43; CI_95%_ =1.53–36.15; Table S13). The Kaplan–Meier curve for OS with the T allele of *VDR* Cdx-2 (rs11568820) is shown in Figure 4 (P_log-rank_ = 0.003). The median OS for the GG genotype was 130.0 (CI_95%_ = 107.6-NR) months and for the AA genotype it was 27.2 (CI_95%_ = 24.3-NR) months, while for the AG genotype it was not reached. Multivariate Cox regression, adjusted for family history of cancer, showed that the *VDR* rs11568820 polymorphism was the only independent factor associated with OS in patients with resected NSCLC (*p* = 0.0129; HR = 7.43; CI_95%_ = 1.53–36.15) (P_likelihood ratio test_ = 0.04) (Table 5).

**Figure 5:**
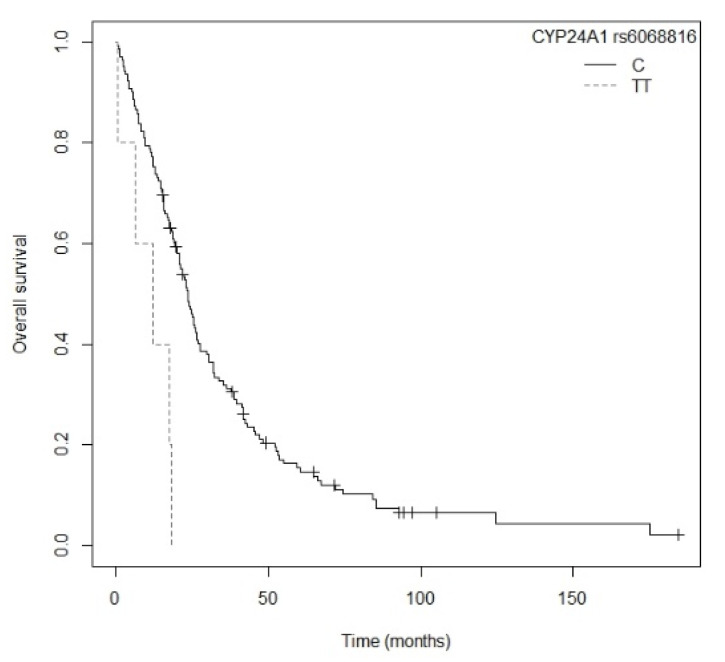
Kaplan–Meier plot of overall survival curves with the C allele of the *CYP24A1* rs6068816 gene polymorphism in the non-resected NSCLC subgroup.

**Figure 6:**
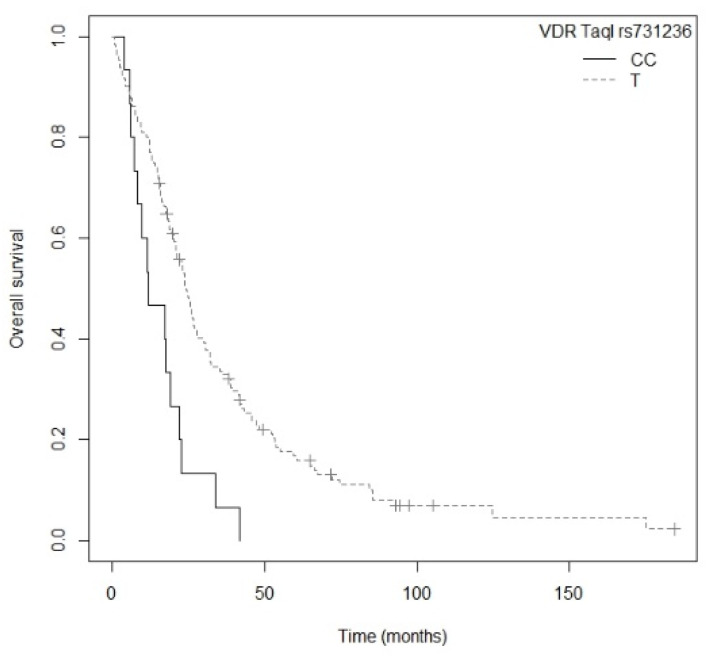
Kaplan–Meier plot of overall survival curves with the T allele of the *VDR* rs731236 gene polymorphism in the non-resected NSCLC subgroup.

In the subgroup of patients with non-resected NSCLC, the TT genotype of the *CYP24A1* rs6068816 polymorphism was associated with higher risk of death in comparison to carriers of the C allele (*p* = 0.0048; HR = 3.75; CI_95%_ = 1.49–9.41; Table S14). Figure 5 shows the Kaplan–Meier plot of OS curves with the C allele for *CYP24A1* rs6068816 (P_log-rank_ = 0.009). Median OS for patients carrying the TT genotype was 12.4 (CI_95%_ = 6.47-NR) months, while for the CC and CT genotypes it was 23.4 (CI_95%_ = 20.1–27.0) and 24.5 (CI_95%_ = 16.0–38.9) months, respectively. On the other hand, three of the five polymorphisms of the *VDR* gene showed an association with OS. Firstly, patients carrying the AA genotype of *VDR* rs1544410 displayed a higher risk of death compared to carriers of the G allele (*p* = 0.0073; HR = 2.08; CI_95%_ = 1.22–3.56; Table S14). Median OS for the AA genotype was 17.5 (CI_95%_ = 9.77–34.1) months, while for the AG and GG genotypes it was 23.7 (CI_95%_ = 16.1–32.0) and 24.5 (CI_95%_ = 21.1–32.2) months, respectively. Figure S16 shows the Kaplan–Meier plot of OS curves with the G allele of *VDR* rs1544410 (P_log-rank_ = 0.020). Secondly, carriers of the AA genotype of *VDR* rs7975232 showed a higher risk of death that those carrying the C allele (*p* = 0.0068; HR = 1.733; CI_95%_ = 1.16–2.58; Table S14). Median OS for the AA genotype was 16.1 (CI_95%_ = 12.3–27.7) months, while for the AC and CC genotypes it was 24.2 (CI_95%_ = 20.1–30.0) and 24.9 (CI_95%_ = 22.1–43.1) months, respectively. Figure S17 shows the Kaplan–Meier plot of OS curves with the C allele of *VDR* rs7975232 (P_log-rank_ = 0.020). Finally, carriers of the CC genotype for *VDR* rs731236 displayed a higher risk of death compared to patients carrying the T allele (*p* = 0.0014; HR = 2.47; CI_95%_ = 1.42–4.28; Table S14). Median OS for the CC genotype was 11.9 (CI_95%_ = 8.30–22.7) months, while for the CT and TT genotypes it was 25.4 (CI_95%_ = 18.3–36.5) and 24.2 (CI_95%_ = 21.1–32.2) months, respectively. Figure 6 shows the Kaplan–Meier plot of OS curves with the T allele for *VDR* rs731236 (P_log-rank_ = 0.004). Cox regression adjusted for gender, drinking status, and tumor stage showed that the *CYP24A1* rs6068816 (*p* = 0.0089; HR = 3.47; CI_95%_ = 1.37–8.79) and *VDR* rs731236 (*p* = 0.0005; HR = 2.71; CI_95%_ = 1.55–4.75) gene polymorphisms were the only independent factors associated with OS in patients with non-resected NSCLC (P_likelihood ratio test_ = 0.000002) (Table 6).

##### Progression-Free Survival

**Figure 7:**
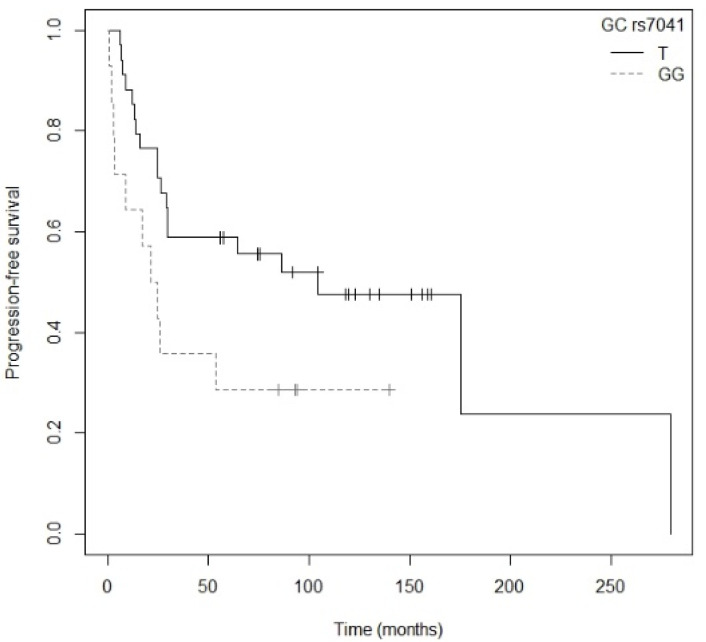
Kaplan–Meier plot of progression-free survival curves with the T allele of the *GC* rs7041 gene polymorphism in the resected NSCLC subgroup.

Patients carrying the AA genotype for the *VDR* rs11568820 polymorphism showed a tendency toward greater progression compared to those with the G allele (*p* = 0.055; HR = 4.35; CI_95%_ = 0.97–19.53; Table S15) in the resected NSCLC subgroup. The Kaplan–Meier plot of PFS curves with the G allele of *VDR* rs11568820 is shown in Figure S18 (P_log-rank_ = 0.040). Patients carrying the GG genotype had a median PFS of 59.0 (CI_95%_ = 24.73-NR) months, while for the AA genotype it was 12.8 (CI_95%_ = 8.67-NR) months and for AG it was not reached. In the case of *GC* rs7041, carriers of the GG genotype exhibited a tendency toward greater risk of progression than those with the TT genotype (*p* = 0.061; HR = 2.122; CI_95%_ = 0.97–4.66; Table S15). The Kaplan–Meier plot of PFS curves with the T allele of GC rs7041 is shown in Figure 7 (P_log-rank_ = 0.060). Patients who carried the TT genotype showed a median PFS of 175.2 (CI_95%_ = 86.10-NR) months, while for the GT and GG genotypes median PFS was 29.6 (CI_95%_ = 24.73-NR) and 23.3 (CI_95%_ = 8.67-NR) months, respectively. Finally, we found that the C allele of the *CYP24A1* rs6068816 polymorphism was associated with a higher risk of progression than the T allele (*p* = 0.0359; HR = 8.49; CI_95%_ = 1.15–62.7; Table S15). However, there were no events for carriers of the TT genotype and for the CT genotype there was a single event, so median follow-up was not reached. This polymorphism was therefore excluded from the multivariate analysis. Cox regression adjusted for tumor stage revealed that the *GC* rs7041 polymorphism was significantly associated with PFS in patients with resected NSCLC (*p* = 0.0447; HR = 2.26; CI_95%_ = 1.02–5.02; Table 7) (P_likelihood ratio test_ = 0.05).

**Figure 8:**
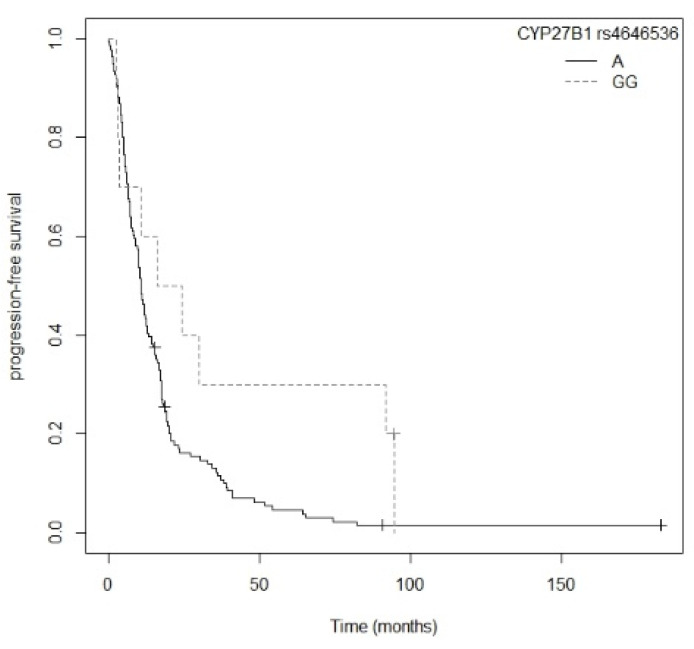
Kaplan–Meier plot of progression-free survival curves with the A allele of the *CYP27B1* rs4646536 gene polymorphism in the non-resected NSCLC subgroup.

**Figure 9:**
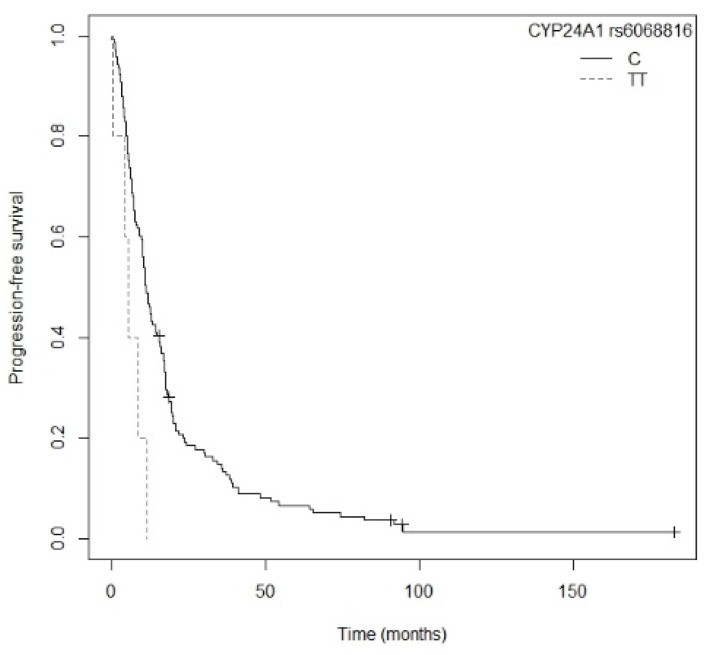
Kaplan–Meier plot of progression-free survival curves with the C allele of the *CYP24A1* rs6068816 gene polymorphism in the non-resected NSCLC subgroup.

**Figure 10:**
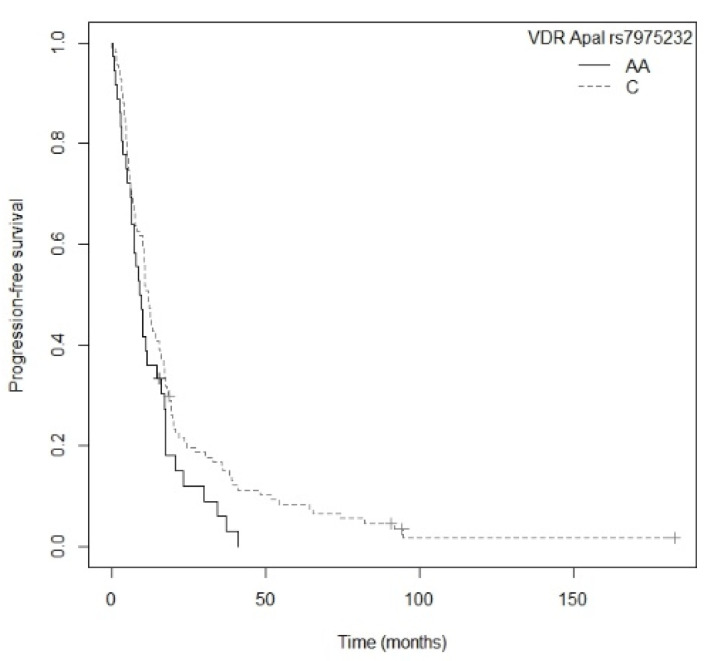
Kaplan–Meier plot of progression-free survival curves with the C allele of the *VDR* rs7975232 gene polymorphism in the non-resected NSCLC subgroup.

A higher risk of progression was shown in non-resected NSCLC patients carrying the A allele for the *CYP27B1* rs4646536 gene polymorphism compared to those with the GG genotype (*p* = 0.0443; HR = 2.05; CI_95% =_ 1.02–4.14; Table S16). The Kaplan–Meier plot of PFS curves with the A allele of *CYP27B1* rs4646536 is shown in Figure 8 (P_log-rank_ = 0.040). Median PFS for carriers of the GG genotype was 20.3 (CI_95%_ = 3.77-NR) months, while for the AG and AA genotypes it was 10.3 (CI_95%_ = 8.5–15.6) and 10.9 (CI_95%_ = 8.37–15.5) months, respectively. Similarly, the G allele of the *CYP27B1* rs3782130 polymorphism displayed a higher risk of progression than the CC genotype (*p* = 0.0452; HR = 2.05; CI_95%_ = 1.01–4.13; Table S16). The Kaplan–Meier plot of PFS curves with the G allele of *CYP27B1* rs3782130 is shown in Figure S19 (P_log-rank_ = 0.040). Patients carrying the CC genotype had a median PFS of 20.3 (CI_95%_ = 3.77-NR) months, while for the GC and GG genotypes median PFS was 11.2 (CI_95%_ = 8.0–17.1) and 10.9 (CI_95%_ = 8.37–15.5) months, respectively. Furthermore, carriers of the G allele of the *CYP27B1* rs10877012 polymorphism showed a higher risk of progression compared to the TT genotype (*p* = 0.0443; HR = 2.05; CI_95%_ = 1.02–4.14; Table S16). Patients with the TT genotype had a median PFS of 20.3 (CI_95%_ = 3.77-NR) months, while for the GT and GG genotypes median PFS was 10.8 (CI_95%_ = 8.0–15.6) and 10.9 (CI_95%_ = 8.37–15.5) months, respectively. The Kaplan–Meier plot of PFS curves with the G allele for *CYP27B1* rs10877012 is shown in Figure S20 (P_log-rank_ = 0.040). Patients carrying the TT genotype of the *CYP27B1* rs6068816 polymorphism also showed greater progression than those carrying the C allele (*p* = 0.0179; HR = 2.99; CI_95%_ = 1.21–7.45; Table S16). The Kaplan–Meier plot of PFS curves with the C allele for *CYP27B1* rs6068816 is shown in Figure 9 (P_log-rank_ = 0.010). Patients with the TT genotype had a median PFS of 5.43 (CI_95%_ = 4.27-NR) months, while for the CC and CT genotypes it was 11.9 (CI_95%_ = 10.1–16.1) and 10.4 (CI_95%_ = 6.20–18.7) months, respectively. Furthermore, patients with the AA genotype for the *VDR* rs7975232 polymorphism displayed a tendency toward greater progression compared to those carrying the C allele (*p* = 0.0643; HR = 1.44; CI_95%_ = 0.98–2.13; Table S16). The Kaplan–Meier plot of PFS curves with the C allele of *VDR* rs7975232 is shown in Figure 10 (P_log-rank_ = 0.060). Patients carrying the AA genotype had a median PFS of 9.47 (CI_95%_ = 6.70–16.1) months, whereas for the AC and CC genotypes it was 11.20 (CI_95%_ = 8.37–16.8) and 12.9 (CI_95%_ = 10.4–17.6) months, respectively. Finally, we found that the CC genotype of the *VDR* rs731236 polymorphism was associated with a higher risk of progression compared to the T allele (*p* = 0.0463; HR = 1.74; CI_95%_ = 1.01–2.99; Table S16). Patients carrying the CC genotype showed a median PFS of 7.1 months (CI_95%_ = 6.20–17.1), while for the CT and TT genotypes it was 10.7 (CI_95%_ = 8.0–16.1) and 12.8 (CI_95%_ = 10.5–17.6) months, respectively. The Kaplan–Meier plot of PFS curves with the T allele of *VDR* rs731236is shown in Figure S21 (P_log-rank_ = 0.040). Cox regression adjusted for BMI showed that the *CYP27B1* rs4646536 (*p* = 0.0411; HR = 2.52; CI_95%_ = 1.04–6.12), *CYP24A1* rs6068816 (*p* = 0.0048; HR = 8.77; CI_95%_ = 1.94–39.7), and *VDR* rs7975232 (*p* = 0.0002; HR = 3.08; CI_95%_ = 1.71–5.54) polymorphisms were significantly associated with PFS in patients with non-resected NSCLC (P_likelihood ratio test_ = 0.00006) (Table 8).

## 4. Discussion

Cancer survival may be influenced by vitamin D through the suppression of cell proliferation, angiogenesis, cell proliferation, and metastasis which means the inhibition of tumor progression. Furthermore, the promotion of apoptosis in cancerous cells could be triggered by vitamin D [5,16]. Survival rates in patients diagnosed with NSCLC vary, even among patients diagnosed with the same stage [9,10,11,12]. Genetic factors may explain these interindividual differences. Several polymorphisms in various genes involved in the vitamin D metabolic pathway have been suggested as possible causes of this variability [5,14,15,31,42,43].

After investigating the potential of the gene polymorphisms involved in the vitamin D metabolic pathway in 194 Caucasian patients (from Spain) with NSCLC, we found that for the general population, patients carrying the A allele for the *CYP27B1* rs4545636 polymorphism had a higher risk of progression and tended to have a higher risk of death than bearers of the GG genotype. Additionally, *CYP27B1* rs4646536 maintained its associated with PFS in the subgroup of non-resected patients. To date there is only one other study, conducted in an Asian population (from China) with 542 NSCLC patients, that has evaluated the influence of *CYP27B1* rs4646536 on survival. However, no statistically significant association was found (*p* = 0.625) [5]. On the other hand, another study carried out in an Asian population (from China), with 153 (NSCLC) tumor samples, where a better overall survival (*p* = 0.018) was associated with a high *CYP27B1* expression. It also found that alteration in gene expression may be due to SNPs, and specifically that the differences in expression were statistically significant in the *CYP27B1* rs3782130 polymorphism (*p* = 0.028) [31]. The importance of expression of *CYP27B1* lies in the fact that it is the only gene capable of converting vitamin D to its active form (1,25-dihydroxycholecalciferol) and it is this product that triggers all the biological functions of vitamin D, after binding to VDR [16]. In our study, the *CYP27B1* rs3782130 polymorphism was associated with PFS in the non-resected patient subgroup in the univariate Cox regression model.

In the resected patient subgroup, the multivariate analysis revealed that patients carrying the AA genotype for *VDR* rs11568820 had a higher risk of death than those with the GG genotype. Previous studies have shown contradictory results. Akiba et al., in a study with 155 Asian NSCLC patients (from Japan), found that the A allele of *VDR* rs11568820 was associated with better overall survival (*p* = 0.04; HR = 0.39; CI_95%_ = 0.16–0.97 for GA/AA vs. GG) [43]. Similarly, another study with 376 patients of Caucasian origin (from the United States) with early stage NSCLC showed that the AA/AG genotypes of *VDR* rs11568820 had better overall survival that the GG genotype (*p* = 0.04; HR = 0.56; CI_95%_ = 0.33–0.95 for GA/AA vs. GG) [15]. However, a study involving 294 Caucasian patients (from the United States) with advanced NSCLC found no association between *VDR* rs11568820 polymorphisms and overall survival (*p* = 0.82) [14]. Moreover, in our study patients bearing the G allele of the *GC* rs7041 polymorphism showed a higher risk of progression than those with the TT genotype. Our findings are in line with the above-mentioned study by Akiba et al., in which the TT genotype of *GC* rs7041 was associated with better PFS (*p* = 0.045; HR = 0.51; CI_95%_ = 0.26–0.99; TT vs. TG/GG) and better OS (*p* = 0.003; HR = 0.21; CI_95%_ = 0.07–0.59; TT vs. TG/GG) [43]. Subsequently, another study was carried out with 542 Asian patients (from China), evaluating the relationship between *GC* rs7041 and survival in NSCLC. However, the results were not statistically significant (*p* = 0.693) [5].

With regard to the subgroup of non-resected patients, our study revealed that a higher risk of death and of progression was associated with the TT genotype for the *CYP24A1* rs6068816 polymorphism in comparison to those carrying the C allele. Only one other study of the relationship between *CYP24A1* rs6068816 and survival has been conducted to date, in 542 Asian patients (from China) with NSCLC. However, it did not find a statistically significant association (*p* = 0.072) [5].

In our study, we also found that in the non-resected patient subgroup the rs7975232 and rs731236 polymorphisms of the *VDR* gene were associated with progression-free survival and overall survival, respectively. Firstly, patients in our study carrying the CC genotype for *VDR* rs731236 had a higher risk of death that those bearing the T allele. Previous studies have reported contradictory results. A study involving 62 patients of Asian origin (from Turkey) with NSCLC showed that the combined TT-CC/TT-TC genotypes for rs731236 and rs2228570, respectively, were associated with worse overall survival (HR = 1.81; CI_95%_ = 1.23–3.48; *p* = 0.04) [51]. However, another later study with 155 Asian patients (from Japan) with NSCLC did not find a statistically significant association between *VDR* rs731236 and overall survival (*p* = 0.26) [43]. The *VDR* rs7975232 polymorphism was associated in our study with a higher risk of death and progression. Specifically, patients carrying the AA genotype showed worse OS and greater progression than those with the C allele. Our results are in line with a previous study in 321 Asian patients (from China) with advanced NSCLC [52]. This found an association between the AA genotype of *VDR* rs7975232 and worse OS (*p* < 0.001; HR = 2.84; CI_95%_ = 2.63–3.94) as well as a strong tendency toward a higher risk of progression (*p* = 0.053; HR = 1.43; CI_95%_ = 0.99–2.78) than with the CC genotype [52]. Moreover, this association has been confirmed in a multiethnic meta-analysis with 3.199 cases in five studies of various types of cancer (prostate, renal, colorectal, lung, and head and neck), which revealed that the AA genotype of *VDR* rs7975232was associated with worse PFS (HR = 1.29; CI_95%_ = 1.02–1.56) [53].

The CYP2R1 rs10741657 polymorphism has been studied recently. A study in an Asian population (from China) with 542 cases of NSCLC found that patients with the GG and AG genotypes had better OS than those with the AA genotype (*p* = 0.033; HR = 0.69; CI_95%_ = 0.46–0.97), particularly elderly patients not receiving chemotherapy [5]. In our patients this effect could not be confirmed. However, we did observe a strong tendency in the univariate Cox regression model toward OS in the general population.

Finally, a multiethnic meta-analysis of various types of cancer with 9926 cases in 10 studies (prostate, lung, colorectal, skin, glioma, and head and neck) for *VDR* rs1544410 and 11,334 cases in 12 studies (breast, prostate, lung, colorectal, skin, glioma, ovarian, and head and neck) for *VDR* rs2228570 evaluated the association of these two polymorphisms with OS. In particular, the AA/AG genotypes of *VDR* rs1544410 were associated with worse OS (HR = 1.40; CI_95%_ = 1.05–1.75; AA/AG vs. GG) [53]. In our study, the univariate Cox regression model showed that the AA genotype for the *VDR* rs1544410 polymorphism was associated with worse OS in non-resected patients compared to the G allele. However, after the multivariate Cox regression this association was not sustained. The *VDR* rs2228570 polymorphism was not associated with OS in the meta-analysis described above (HR = 1.26; CI_95%_ = 0.96–1.56) [53]. Our study is in agreement with those results, since we did not obtain statistically significant associations for *VDR* rs2228570 in any of the subgroups analyzed.

Vitamin D is strongly related to survival and risk of NSCLC. There is therefore a presumption that alterations in the genes involved in the metabolic pathway of vitamin D may affect their expression and functionality [15,28,36,37,38,39,40]. Each of the genes fulfills a unique and characteristic function within the metabolic process of vitamin D. We can therefore surmise that variations in one or more genes may entail a worse prognosis. Thus far, we know that the genetic expression of *CYP24A1*, *CYP27B1*, and *VDR* in lung cancer is affected by tumor differentiation and characterization. When the tumor is poorly differentiated, there is greater expression of *CYP24A1* and reduced expression of *CYP27B1* [36,38]. Increased expression of *VDR* in lung cancer is associated with better survival [40,54]. This may be related to a lower proliferative state and G1-phase arrest of high VDR-expressing tumor cells [40,54]. Continued research is needed on the mechanism by which the SNPs associated with the vitamin D metabolic pathway affect overall survival and progression-free survival in patients with NSCLC.

This study presents a cohort of patients diagnosed with NSCLC from the same institution and treated under the same therapeutic protocol, ensuring the uniformity of the sample in the measurement of the survival variables analyzed. Although the sample size in our study is limited and it was not possible to detect some associations, the effect of the polymorphisms in the *CYP27B1*, *CYP24A1*, *GC* y *VDR* genes was clear.

## 5. Conclusions

This study found that the *CYP27B1* rs4646535, *CYP24A1* rs6068816, *GC* rs7041, *VDR* rs11568820, *VDR* rs731236, and *VDR* rs7975232 polymorphisms are associated with survival in NSCLC and may have a substantial role as prognostic markers of the disease. We found no relationship between the *CYP27B1* rs3782130, *CYP27B1* rs10877012, *CYP24A1* rs4809957, *CYP2R1* rs10741657, *VDR* rs1544410, and *VDR* rs2228570 polymorphisms and survival in our patients.

## Figures and Tables

**Table 1:** Gene polymorphisms and TaqMan^®^ ID.

Gene	dbSNP ID	Assay ID
VDR	rs1544410 (BsmI)	AN324M4 *
	rs11568820 (Cdx-2)	C___2880808_10
	rs2228570 (FokI)	C__12060045_20
	rs7975232 (ApaI)	C__28977635_10
	rs731236 (TaqI)	C___2404008_10
CYP27B1	rs4646536	C__25623453_10
	rs3782130	ANGZRHH *
	rs10877012	C__26237740_10
CYP24A1	rs6068816	C__25620091_20
	rs4809957	C___3120981_20
GC	rs7041	C___3133594_30
CYP2R1	rs10741657	C___2958430_10

* The polymorphisms were analyzed using custom assays by ThermoFisher Scientific (Waltham, MA, USA).

**Table 2:** Classification of the 194 NSCLC patients according to the clinico-pathologic characteristics.

	n	%
Gender		
Female	53	27.32
Male	141	72.68
Family history of cancer		
Yes	102	53.4
No	89	46.6
Previous lung disease		
Yes	55	28.35
No	139	71.65
Smoking status		
Current smokers	92	47.42
Former smokers	75	38.66
Non smokers	27	13.92
Alcoholic status		
Current drinkers	33	20.50
Former drinkers	4	2.48
Non-drinkers	124	77.02
BMI	27.09 ± 5.12	
<24	31	27.19
>24	83	72.81
Age at NSCLC diagnosis	60.86 ± 10.51	
≤60	85	43.81
>60	109	56.19
Histology		
Adenocarcinoma	119	61.34
Squamous cell carcinoma	72	37.11
Unknown	3	1.55
Tumor stage		
I, II or IIIA	63	32.64
IIIB or IV	130	67.36
First course of treatment		
Surgery	48	24.74
Chemoradiotherapy	121	62.37
Targeted therapy	25	12.89
Survival		
PFS	13.7 [6.46–29.96]	
OS	26.8 [15.5–64.2]	

Qualitative variables: number (percentage); Quantitative variables: Normal distribution: mean ± standard deviation; Non-normal distribution: P50 (P25, P75).

**Table 3:** Influence of gene polymorphisms and clinical characteristic on overall survival of 194 NSCLC patients.

	Overall Survival	
	HR (CI_95%_)	*p*-Value
Gender (Male)	1.63 (1.11–2.38)	0.0119
Tumor stage (IIIB-IV)	2.25 (1.25–4.07)	0.0070
First-line treatment (No surgery)	2.65 (1.31–5.35)	0.0064
*CYP27B1* rs4646536-A	2.01 (0.98–4.14)	0.0569

HR = hazard ratio. CI_95%_: 95% confidence interval.

**Table 4:** Influence of gene polymorphisms and clinical characteristics on progression-free survival of 194 NSCLC patients.

	Progression-Free Survival	
	HR (CI_95%_)	*p*-Value
First-line treatment (no surgery)	3.89 (2.54–5.96)	<0.001
*CYP27B1* rs4646536_A	2.11 (1.11–4.04)	0.0233

HR = hazard ratio. CI_95%_: 95% confidence interval.

**Table 5:** Influence of clinical characteristic and gene polymorphisms on overall survival of resected NSCLC patients.

	Overall Survival	
	HR (CI_95%_)	*p*-Value
*VDR* rs11568820_AA	7.43 (1.53–36.15)	0.0129

HR = hazard ratio. CI_95%_: 95% confidence interval.

**Table 6:** Influence of gene polymorphisms and clinical characteristic on overall survival of non-resected NSCLC patients.

	Overall Survival	
	HR (CI_95%_)	*p*-Value
Gender (Male)	1.67 (1.11–2.52)	0.0142
Tumor stage (IIIB—IV)	2.42 (1.29–4.51)	0.0055
*CYP24A1* rs6068816_TT	3.47 (1.37–8.79)	0.0089
*VDR* rs731236_CC	2.71 (1.55–4.75)	0.0005

HR = hazard ratio. CI_95%_: 95% confidence interval.

**Table 7:** Influence of gene polymorphisms and clinical characteristic on progression-free survival of resected NSCLC patients.

	Progression-Free Survival	
	HR (CI_95%_)	*p*-Value
Estadio (IIIB-IV)	11.8 (1.34–104.1)	0.0262
*GC* rs7041_GG	2.26 (1.02–5.02)	0.0447

HR = hazard ratio. CI_95%_: 95% confidence interval.

**Table 8:** Influence of gene polymorphisms and clinical characteristic on progression-free survival of non-resected NSCLC patients.

	Progression-Free Survival	
	HR (CI_95%_)	*p*-Value
BMI ( > 24)	2.23 (1.27–3.89)	0.0051
*CYP27B1* rs4646536_A	2.52 (1.04–6.12)	0.0411
*CYP24A1* rs6068816_TT	8.77 (1.94–39.7)	0.0048
*VDR* rs7975232_AA	3.08 (1.71–5.54)	0.0002

HR = hazard ratio. CI_95%_: 95% confidence interval.

## Supplementary Materials

The following are available online at https://www.mdpi.com/article/10.3390/nu13113783/s1, Table S1: Clinical characteristics and association with overall survival of the 194 NSCLC patients. Table S2: Clinical characteristics and association with progression-free survival of the 194 NSCLC patients. Table S3: Clinical characteristics and association with overall survival of the resected NSCLC patients. Table S4: Clinical characteristics and association with progression-free survival of the resected NSCLC patients. Table S5: Clinical characteristics and association with overall survival of the non-resected NSCLC patients. Table S6: Clinical characteristics and association with progression-free survival of the non-resected NSCLC patients. Table S7: Minor allele frequencies of SNPs. Table S8: Hardy–Weinberg Equilibrium (OS). Table S9: Hardy–Weinberg Equilibrium (PFS). Table S10: Linkage disequilibrium. Table S11: Polymorphisms and association with overall survival of the 194 NSCLC patients. Table S12: Polymorphisms and association with progression-free survival of the 194 NSCLC patients. Table S13: Polymorphisms and association with overall survival of the resected NSCLC patients. Table S14: Polymorphisms and association with overall survival of the non-resected NSCLC patients. Table S15: Polymorphisms and association with progression-free survival of the resected NSCLC patients. Table S16: Polymorphisms and association with progression-free survival of the non-resected NSCLC patients. Figure S1. Kaplan–Meier plot of overall survival curves by gender in 194 patients with NSCLC. Figure S2. Kaplan–Meier plot of overall survival curves by drinking status in 194 patients with NSCLC. Figure S3. Kaplan–Meier plot of overall survival curves by tumor stage in 194 patients with NSCLC. Figure S4. Kaplan–Meier plot of overall survival curves by first-line treatment (divided by surgery) in 194 patients with NSCLC. Figure S5. Kaplan–Meier plot of progression-free survival curves with previous lung disease in 194 patients with NSCLC. Figure S6. Kaplan–Meier plot of progression-free survival curves by drinking status in 194 patients with NSCLC. Figure S7. Kaplan–Meier plot of progression-free survival curves by tumor stage in 194 patients with NSCLC. Figure S8. Kaplan–Meier plot of progression-free survival curves by first-line treatment (divided by surgery) in 194 patients with NSCLC. Figure S9. Kaplan–Meier plot of overall survival curves with family history of cancer in the resected NSCLC subgroup. Figure S10. Kaplan–Meier plot of progression-free survival curves by tumor stage in the resected NSCLC subgroup. Figure S11. Kaplan–Meier plot of overall survival curves by gender in the non-resected NSCLC subgroup. Figure S12. Kaplan–Meier plot of overall survival curves by drinking status in the non-resected NSCLC subgroup. Figure S13. Kaplan–Meier plot of overall survival curves by tumor stage in the non-resected NSCLC subgroup. Figure S14. Kaplan–Meier plot of progression-free survival curves by body mass index in the non-resected NSCLC subgroup. Figure S15. Kaplan–Meier plot of overall survival curves with the T allele of the GC rs7041 gene polymorphism in the resected NSCLC subgroup. Figure S16. Kaplan–Meier plot of overall survival curves with the G allele of the *VDR* rs1544410 gene polymorphism in the non-resected NSCLC subgroup. Figure S17. Kaplan–Meier plot of overall survival curves with the C allele of the *VDR* rs7975232 gene polymorphism in the non-resected NSCLC subgroup. Figure S18. Kaplan–Meier plot of progression-free survival curves with the G allele of the *VDR* rs11568820 gene polymorphism in the resected NSCLC subgroup. Figure S19. Kaplan–Meier plot of progression-free survival curves with the G allele of the *CYP27B1* rs3782130 gene polymorphism in the non-resected NSCLC subgroup. Figure S20. Kaplan–Meier plot of progression-free survival curves with the G allele of the *CYP27B1* rs10877012 gene polymorphism in the non-resected NSCLC subgroup. Figure S21. Kaplan–Meier plot of progression-free survival curves with the T allele of the *VDR* rs731236 gene polymorphism in the non-resected NSCLC subgroup.

## Abbreviations

AJCC: American Joint Committee on Cancer; CI: confidence interval; *CYP24A1*: cytochrome P450 family 24 subfamily A member 1; *CYP27B1*: cytochrome P450 family 27 subfamily B member 1; *CYP2R1*: cytochrome P450 family 2 subfamily R member 1; *GC*: GC Vitamin D Binding Protein (gene); HR: hazard ratio; BMI: body mass index; MAF: minor allele frequency; NA: not available; NR: not reached; NSCLC: non-small-cell lung cancer; OS: overall survival; PCR: polymerase chain reaction; PFS: progression-free survival; SAS: Sistema Andaluz de Salud (Andalusian Health Service); SNP: single nucleotide polymorphism; VDBP: vitamin D binding protein; *VDR*: vitamin D receptor (gene);VDR: vitamin D receptor (protein); RXR: retinoid X receptor.
