# Supplementary material for: Impact of Genetic Polymorphisms on the Metabolic Pathway of Vitamin D and Survival in Non-Small Cell Lung Cancer
Source: Nutrients. 2021 Oct 25;13(11):3783. doi: 10.3390/nu13113783 (PMC8621267; doi:10.3390/nu13113783)
Supplement: Supplementary file 1 [file nutrients-13-03783-s001.zip › Supplementary Files/Table S1.pdf]

Table S1. Clinical characteristics and association with overall survival of the 194 NSCLC patients.

Characteristic	OS								
	N	Events	MST (mo)	IC95%	Log-Rank p-value	Reference Category	Univariate Cox Model		
							HR	IC95%	p-value
Gender									
Female	53	36	38.9	25.6-124.5	0.030	Female	1.505	1.04-2.19	0.0324
Male	141	118	26.1	22.1-32.2					
Family history									
Yes	102	88	30.4	25.4-38.9	0.500				
No	89	65	24.9	20.1-41.8					
Previous lung disease									
Yes	55	39	32.2	23.4-75.0	0.100				
No	139	115	26.9	23.8-36.5					
Smoking status									
Current smokers	92	76	27.9	23.7-45.9	1.000				
Former smokers	75	58	26.4	20.1-42.5					
Non-smokers	27	20	26.9	24.5-48.4					
Alcoholic status									
Current drinkers	33	29	21.43	13.03-32.0	0.0005	Non-drinkers	1.772	1.16-2.70	0.0079
Former drinkers	4	4	8.95	5.87-NR			4.487	1.62-12.43	0.0039
Non-drinkers	124	91	36.53	27.03-48.4			1		
Age at NSCLC diagnosis									
≤60	85	65	27.7	22.7-42.5	0.300				
>60	109	89	30.1	24.5-41.7					
BMI									
<24	31	20	53.8	32.0-NR	**0.080**	<24	1.563	0.94-2.59	0.0843
>24	83	63	35.2	24.5-55					
Histology									
Adenocarcinoma	119	98	26.1	23.7-32.2	**0.070**	Squamous cell carcinoma	1.363	0.98- 1.91	0.0694
Squamous cell carcinoma	72	54	37.7	22.1-64.7					
Tumor stage									
I, II and IIIA	63	33	107.6	72.0-NR	<0.001	I, II and IIIA	4.414	2.95-6.59	<0.001
IIIB and IV	130	120	21.1	17.8-24.9					
First course of treatment									
Surgery	48	22	130.0	102.5-NR	<0.001	Surgery	1		
Chemoradiotherapy	121	110	22.7	18.4-27.0			5.027	3.12-8.10	<0.001
Targeted therapy	25	22	20.1	20.1-41.8			5.709	3.07-10.62	<0.001
First course of treatment (divided by surgery)									
Surgery	48	22	130.0	102.5-NR	<0.001	Surgery	5.108	3.18- 8.19	<0.001
No surgery	146	132	23.2	19.5-26.4					

MST: median survival time (months)
NR: not reached.
HR: hazard ratio
IC95%: 95% confidence Interval
BMI: body mass index
