# Supplementary material for: Impact of Genetic Polymorphisms on the Metabolic Pathway of Vitamin D and Survival in Non-Small Cell Lung Cancer
Source: Nutrients. 2021 Oct 25;13(11):3783. doi: 10.3390/nu13113783 (PMC8621267; doi:10.3390/nu13113783)
Supplement: Supplementary file 1 [file nutrients-13-03783-s001.zip › Supplementary Files/Table S10.pdf]

Table S10. Linkage disequilibrium.

**Chr**	**BP**	**SNP**	**Chr**	**BP**	**SNP**	**R2**	**D'**
12	47844974	rs731236	12	47845054	rs7975232	0.512229	0.938
12	47844974	rs731236	12	47846052	rs1544410	0.940695	1.000
12	47845054	rs7975232	12	47846052	rs1544410	0.540406	0.941
12	57764205	rs4646536	12	57768115	rs3782130	0.949924	1.000
12	57764205	rs4646536	12	57768302	rs10877012	0.950085	1.000
12	57768115	rs3782130	12	57768302	rs10877012	1	1.000
Chr: Chromosome; BP: Physical position (base-pair)							
