# Supplementary material for: Impact of Genetic Polymorphisms on the Metabolic Pathway of Vitamin D and Survival in Non-Small Cell Lung Cancer
Source: Nutrients. 2021 Oct 25;13(11):3783. doi: 10.3390/nu13113783 (PMC8621267; doi:10.3390/nu13113783)
Supplement: Supplementary file 1 [file nutrients-13-03783-s001.zip › Supplementary Files/Table S11.pdf]

Table S11. Polymorphisms and association with overall survival of the 194 NSCLC patients.

Gene	SNPs	Genotype	N	OS							
				Events	MST (mo)	IC95%	Log-Rank p-value	Ref Cat	Univariate Cox Model		
									HR	IC95%	p-value
CPY27B1	rs4646536	AA	114	95	27.0	23.2-41.8	0.060				
		AG	65	51	24.9	22.7-38.0					
		GG	15	8	92.9	34.1-NR					
		A	179	146	26.4	23.7-32.4	0.020	G	2.318	1.13-4.73	0.0211
		G	80	59	28.1	23.8-41.6	0.500				
	rs3782130	CC	13	8	41.8	32.0-NR	0.200				
		GC	64	49	26.4	23.4-39.1					
		GG	116	96	27.0	23.2-41.8					
		C	77	57	28.1	24.3-41.6	0.500				
		G	180	145	26.7	23.9-33.8	0.100				
	rs10877012	TT	13	8	41.8	32.0-NR	0.200				
		GT	65	50	26.1	23.4- 39.1					
		GG	116	96	27.0	23.2- 41.8					
		T	78	58	28.1	23.8- 41.6	0.500				
		G	181	146	26.7	23.8- 33.8	**0.090**	T	1.826	0.89-3.73	0.0988
CYP24A1	rs6068816	CC	147	119	30.0	24.50-39.6	0.900				
		CT	40	30	27.7	22.70-52.2					
		TT	7	5	17.6	6.47-NR					
		C	187	149	28.1	24.50-38	0.800				
		T	47	35	26.9	19.0-43.1	1.000				
	rs4809957	GG	9	5	60.7	26.9-NR	0.300				
		GA	63	53	25.4	20.9-41.7					
		AA	122	96	30.0	23.4-38.0					
		G	72	58	26.7	23.8-41.8	0.900				
		A	185	149	27.0	23.9-35.2	0.200				
GC	rs7041	TT	41	29	39.6	22.1-114.4	0.300				
		TG	89	71	25.4	23.2- 42.5					
		GG	64	54	26.7	21.1-36.5					
		T	130	100	30.0	23.9-42.5	0.300				
		G	153	125	26.1	23.7-33.8	0.100				
CYP2R1	rs10741657	GG	75	58	27.9	21.0-45.9	0.100				
		GA	93	73	35.2	24.9-45.9					
		AA	24	21	23.4	19.5-38.0					
		G	168	131	30.8	24.9-41.7	**0.050**	G	1.585	0.99-2.52	0.0525
		A	117	94	30.8	24.3-41.6	0.400				
VDR	rs1544410 (BsmI)	AA	24	18	22.1	17.5-54.3	0.500				
		AG	102	76	30.7	24.2-45.9					
		GG	68	60	26.1	23.8-42.5					
		A	126	94	30.1	23.1-41.6	0.400				
		G	170	136	28.1	24.5-38.9	0.600				
	rs11568820 (Cdx-2)	AA	11	10	24.3	22.1-NR	0.400				
		AG	69	53	26.1	21.0-45.3					
		GG	114	91	30.0	24.5-43.1					
		A	80	63	25.4	22.7-38.0	0.500				
		G	183	144	27.9	24.5-39.1	0.200				
	rs2228570 (FokI)	CC	85	72	26.7	23.4-41.6	0.600				
		CT	86	64	30.7	23.8-45.9					
		TT	23	18	24.9	22.1-47.4					
		C	171	136	27.9	24.3-38.0	0.600				
		T	109	82	30.1	23.8-41.8	0.500				
	rs7975232 (ApaI)	AA	48	39	22.7	17.5-39.6	0.400				
		AC	93	74	26.9	23.7-38.9					
		CC	53	41	39.1	24.5-64.7					
		A	141	113	26.7	22.7-32.2	0.400				
		C	146	115	30.0	24.9-42.5	0.200				
	rs731236 (TaqI)	CC	21	17	19.0	11.6-54.3	0.200				
		CT	103	75	32.2	26.4-52.6					
		TT	70	62	25.8	23.8-39.1					
		C	124	92	30.7	23.1-41.7	0.300				
		T	173	137	30.0	24.9-39.1	0.300				

MST: median survival time (months)
NR: not reached
Ref Cat: reference category
HR: hazard ratio
IC95%: 95% confidence interval
