# Supplementary material for: Impact of Genetic Polymorphisms on the Metabolic Pathway of Vitamin D and Survival in Non-Small Cell Lung Cancer
Source: Nutrients. 2021 Oct 25;13(11):3783. doi: 10.3390/nu13113783 (PMC8621267; doi:10.3390/nu13113783)
Supplement: Supplementary file 1 [file nutrients-13-03783-s001.zip › Supplementary Files/Table S12.pdf]

Table S12. Polymorphisms and association with progression-free survival of the 194 NSCLC patients.

Gene	SNPs	Genotype	N	PFS							
				Events	MST (mo)	IC95%	Log-Rank p-value	Ref Cat	Univariate Cox Model		
									HR	IC95%	p-value
CPY27B1	rs4646536	AA	114	103	13.3	10.93-17.6	0.070				
		AG	65	56	12.3	9.17-17.6					
		GG	15	11	30.0	12.30-NR					
		A	179	159	13.0	10.9-16.8	0.020	GG	2.069	1.08-3.93	0.0266
		G	80	67	14.2	10.3-17.9	0.600				
	rs3782130	CC	13	11	24.5	10.7-NR	0.400				
		GC	64	53	14.2	10.2-17.9					
		GG	116	105	13.1	10.9-17.6					
		C	77	64	14.6	10.7-18.7	0.400				
		G	180	158	13.3	11.0-17.1	0.200				
	rs10877012	TT	13	11	24.5	10.7-NR	0.400				
		GT	65	54	14.2	10.1-17.9					
		GG	116	105	13.1	10.9-17.6					
		T	78	65	14.4	10.7-18.7	0.400				
		G	181	159	13.2	11.0-17.1	0.200				
CYP24A1	rs6068816	CC	147	131	14.2	11.93-17.6	1.000				
		CT	40	34	11.9	9.07-21.6					
		TT	7	5	8.5	4.27-NR					
		C	187	165	14.2	11.87-17.1	0.900				
		T	47	39	11.2	8.5-19.6	0.800				
	rs4809957	GG	9	6	36.1	13.5-NR	0.200				
		GA	63	56	15.9	10.2-19.6					
		AA	122	108	13.0	10.9-16.7					
		G	72	62	17.0	10.7-20.2	0.400				
		A	185	164	13.0	11.0-16.8	**0.080**	G	2.03	0.89-4.59	0.0891
GC	rs7041	TT	41	32	15.6	10.2-37.1	0.300				
		TG	89	80	13.2	10.5-17.6					
		GG	64	58	14.4	10.7-18.7					
		T	130	112	13.3	10.9-17.7	0.500				
		G	153	138	13.5	11.0-17.1	0.100				
CYP2R1	rs10741657	GG	75	64	14.2	10.07-19.2	0.300				
		GA	93	81	13.9	10.93-18.7					
		AA	24	23	12.7	9.07-21.8					
		G	168	145	14.1	11.87-17.5	0.100				
		A	117	104	13.9	11.0-17.6	0.500				
VDR	rs154410 (BsmI)	AA	24	19	15.0	9.07-30.0	0.900				
		AG	102	88	12.5	10.50-17.5					
		GG	68	63	15.6	11.17-20.0					
		A	126	107	13.1	10.2-17.1	0.700				
		G	170	151	13.3	11.20-17.1	0.700				
	rs11568820 (Cdx-2)	AA	11	11	16.1	13.2-NR	0.600				
		AG	69	59	10.7	7.7-17.6					
		GG	114	100	15.6	12.0-19.2					
		A	80	70	11.9	10.0-17.1	0.400				
		G	183	159	13.0	11.0-17.1	0.400				
	rs2228570 (FokI)	CC	85	76	14.6	11.2-17.7	0.600				
		CT	86	72	12.9	10.3-18.7					
		TT	23	22	12.7	10.9-24.9					
		C	171	148	14.2	10.9-17.5	0.400				
		T	109	94	12.7	10.7-17.9	0.900				
	rs7975232 (ApaI)	AA	48	42	11.8	9.07-17.7	0.600				
		AC	93	80	13.5	10.50-18.4					
		CC	53	48	15.6	10.97-21.8					
		A	141	122	12.8	10.5-17.5	0.800				
		C	146	128	14.2	11.87-17.6	0.300				
	rs731236 (TaqI)	CC	21	17	10.0	6.7-30.0	0.900				
		CT	103	88	13.5	10.7-17.7					
		TT	70	65	15.6	11.2-19.4					
		C	124	105	13.1	10.2-17.1	0.600				
		T	173	153	14.2	11.4-17.6	1.000				

MST: median survival time (months)
NR: not reached
Ref Cat: reference category
HR: hazard ratio
IC95%: 95% confidence interval
