# Supplementary material for: Impact of Genetic Polymorphisms on the Metabolic Pathway of Vitamin D and Survival in Non-Small Cell Lung Cancer
Source: Nutrients. 2021 Oct 25;13(11):3783. doi: 10.3390/nu13113783 (PMC8621267; doi:10.3390/nu13113783)
Supplement: Supplementary file 1 [file nutrients-13-03783-s001.zip › Supplementary Files/Table S13.pdf]

Table S13. Polymorphisms and association with overall survival of the resected NSCLC patients.

Gene	SNPs	Genotype	N	OS							
				Events	MST (mo)	IC95%	Log-Rank p-value	Ref Cat	Univariate Cox Model		
									HR	IC95%	p-value
CPY27B1	rs4646536	AA	29	17	108	64.7-NR	**0.080**				
		AG	14	5	NR	102.5-NR					
		GG	5	0	NR	NR-NR					
		A	43	22	126	88.6-NR	**0.050**				
		G	19	5	NR	114.4-NR	**0.060**	G	2.54	0.93-6.89	0.0676
	rs3782130	CC	3	0	NR	NR-NR	0.100				
		GC	15	5	NR	102.5-NR					
		GG	30	17	126	64.7-NR					
		C	18	5	NR	114.4-NR	**0.070**	C	2.42	0.89-6.59	0.0827
		G	45	22	126	103-NR	0.100				
	rs10877012	TT	3	0	NR	NR-NR	0.100				
		GT	15	5	NR	102.5-NR					
		GG	30	17	126	64.7-NR					
		T	18	5	NR	114.4-NR	**0.070**	T	2.42	0.89-6.58	0.0827
		G	45	22	126	103-NR	0.100				
CYP24A1	rs6068816	CC	39	21	114	75-NR	0.200				
		CT	7	1	NR	NR-NR					
		TT	2	0	NR	NR-NR					
		C	46	22	126	103-NR	0.300				
		T	9	1	NR	NR-NR	**0.080**	T	4.99	0.67-37.2	0.117
	rs4809957	GG	5	1	NR	NR-NR	0.700				
		GA	15	8	114	75.0-NR					
		AA	28	13	176	88.6-NR					
		G	20	9	130	107.6-NR	0.800				
		A	43	21	126	103-NR	0.500				
GC	rs7041	TT	12	3	176.1	126.4-NR	0.030				
		TG	22	10	107.6	75.0-NR					
		GG	14	9	61.2	24.3-NR					
		T	34	13	176.1	114.4-NR	0.020	T	2.72	1.14-6.47	0.0242
		G	36	19	108	54.3-NR	0.050				
CYP2R1	rs10741657	GG	23	12	126.4	64.7-NR	0.800				
		GA	23	9	130.0	107.6-NR					
		AA	2	1	24.3	24.3-NR					
		G	46	21	130.0	102.5-NR	0.900				
		A	25	10	130	107.6-NR	0.600				
VDR	rs1544410 (BsmI)	AA	7	2	NR	54.3-NR	0.600				
		AG	27	11	130	102.5-NR					
		GG	14	9	108	64.7-NR					
		A	34	13	130	102.5-NR	0.400				
		G	41	20	126	102.5-NR	0.600				
	rs11568820 (Cdx-2)	AA	2	2	27.2	24.3-NR	0.010				
		AG	16	5	NR	102.5-NR					
		GG	30	15	126.4	107.6-NR					
		A	18	7	NR	88.6-NR	0.900				
		G	46	20	130.0	107.6-NR	0.003	G	7.434	1.53-36.15	0.0129
	rs2228570 (FokI)	CC	21	10	126	75.0-NR	1.000				
		CT	22	10	114	88.6-NR					
		TT	5	2	130	130.0-NR					
		C	43	20	126	103-NR	0.900				
		T	27	12	130	88.6-NR	0.900				
	rs7975232 (ApaI)	AA	12	5	103	75.0-NR	1.000				
		AC	22	10	130	45.0-NR					
		CC	14	7	126	88.6-NR					
		A	34	15	130	102.5-NR	0.900				
		C	36	17	130	108-NR	1.000				
	rs731236 (TaqI)	CC	6	2	NR	54.3-NR	0.700				
		CT	28	11	130	102.5-NR					
		TT	14	9	108	64.7-NR					
		C	34	13	130	102.5-NR	0.400				
		T	42	20	126	102.5-NR	0.800				

MST: median survival time (months)
NR: not reached
Ref Cat: reference category
HR: hazard ratio
IC95%: 95% confidence interval
Inf: infinite
