# Supplementary material for: Impact of Genetic Polymorphisms on the Metabolic Pathway of Vitamin D and Survival in Non-Small Cell Lung Cancer
Source: Nutrients. 2021 Oct 25;13(11):3783. doi: 10.3390/nu13113783 (PMC8621267; doi:10.3390/nu13113783)
Supplement: Supplementary file 1 [file nutrients-13-03783-s001.zip › Supplementary Files/Table S14.pdf]

Table S14. Polymorphisms and association with overall survival of the non-resected NSCLC patients.

Gene	SNPs	Genotype	N	OS					Univariate Cox Model		
				Events	MST (mo)	IC95%	Log-Rank p-value	Ref Cat	HR	IC95%	p-value
CPY27B1	rs4646536	AA	85	78	22.1	18.8-27.9	0.200				
		AG	51	46	22.7	17.1-26.9					
		GG	10	8	36.5	11.9-NR					
		A	136	124	22.7	19.0-25.8	0.100				
		G	61	54	23.7	18.3-32.0	0.900				
	rs3782130	CC	10	8	36.5	11.9-NR	0.200				
		GC	49	44	23.4	17.1-27.7					
		GG	86	79	22.4	18.8-27.0					
		C	59	52	23.8	18.3-32.2	1.000				
		G	135	123	22.7	19.0-25.8	0.100				
	rs10877012	TT	10	8	36.5	11.9-NR	0.200				
		GT	50	45	23.4	16.1-27.7					
		GG	86	79	22.4	18.8-27.0					
		T	60	53	23.8	18.3-32.0	1.000				
		G	136	124	22.7	19.0-25.8	0.100				
CYP24A1	rs6068816	CC	108	98	23.4	20.1-27.0	0.009				
		CT	33	29	24.5	16.0-38.9					
		TT	5	5	12.4	6.47-NR					
		C	141	127	23.7	20.9-27.0	0.002	C	3.752	1.49-9.41	0.0048
		T	38	34	21.1	15.5-30.7	0.800				
	rs4809957	GG	4	4	28.8	15.2-NR	0.700				
		GA	48	45	20.9	17.5-26.7					
		AA	94	83	23.1	18.8-32.0					
		G	52	49	23.7	17.5-26.9	0.500				
		A	142	128	23.1	19.0-26.1	0.900				
GC	rs7041	TT	29	26	22.1	17.1-39.6	0.700				
		TG	67	61	22.7	16.2-25.4					
		GG	50	45	25.8	18.8-35.2					
		T	96	87	22.1	17.8-25.4	0.400				
		G	117	106	23.4	19.0-26.4	0.800				
CYP2R1	rs10741657	GG	52	46	20.5	17.8-27.9	0.700				
		GA	70	64	24.9	20.1-39.1					
		AA	22	20	23.2	19.5-38.0					
		G	122	110	23.7	19.0-27.9	0.400				
		A	92	84	24.2	21.4-34.1	0.700				
VDR	rs1544410 (BsmI)	AA	17	16	17.5	9.77-34.1	0.020				
		AG	75	65	23.7	16.1-32.0					
		GG	54	51	24.5	21.1-32.2					
		A	92	81	21.0	16.1-26.9	0.700				
		G	129	116	24.2	21.0-27.7	0.006	G	2.082	1.22-3.56	0.0073
	rs11568820 (Cdx-2)	AA	9	8	24.2	16.0-NR	0.700				
		AG	53	48	21.0	13.0-27.0					
		GG	84	76	23.7	20.1-30.8					
		A	62	56	22.1	16.0-27.0	0.400				
		G	137	124	23.1	19.0-26.4	0.800				
	rs2228570 (FokI)	CC	64	62	23.1	18.4-26.9	0.700				
		CT	64	54	23.2	16.2-38.0					
		TT	18	16	23.1	20.1-42.5					
		C	128	116	23.2	18.7-26.9	0.500				
		T	82	70	23.2	19.0-30.8	0.300				
	rs7975232 (ApaI)	AA	36	34	16.1	12.3-27.7	0.020				
		AC	71	64	24.2	20.1-30.0					
		CC	39	34	24.9	22.1-43.1					
		A	107	98	21.1	17.5-26.4	0.400				
		C	110	98	24.5	21.4-30.0	0.006	C	1.733	1.16-2.58	0.0068
	rs731236 (TaqI)	CC	15	15	11.9	8.30-22.7	0.004				
		CT	75	64	25.4	18.3-36.5					
		TT	56	53	24.2	21.1-32.2					
		C	90	79	21.0	16.1-27.0	0.900				
		T	131	117	24.2	21.0-27.9	0.0009	T	2.466	1.42-4.28	0.00138

MST: median survival time (months)
NR: not reached
Ref Cat: reference category
HR: hazard ratio
IC95%: 95% confidence interval
