# Supplementary material for: Impact of Genetic Polymorphisms on the Metabolic Pathway of Vitamin D and Survival in Non-Small Cell Lung Cancer
Source: Nutrients. 2021 Oct 25;13(11):3783. doi: 10.3390/nu13113783 (PMC8621267; doi:10.3390/nu13113783)
Supplement: Supplementary file 1 [file nutrients-13-03783-s001.zip › Supplementary Files/Table S15.pdf]

Table S15. Polymorphisms and association with progression-free survival of the resected NSCLC patients.

Gene	SNPs	Genotype	N	PFS							
				Events	MST (mo)	IC95%	Log-Rank p-value	Ref Cat	Univariate Cox Model		
									HR	IC95%	p-value
CPY27B1	rs4646536	AA	29	19	29.8	24.9-NR	0.300				
		AG	14	8	57.8	16.8-NR					
		GG	5	2	279.2	NR-NR					
		A	43	27	29.8	24.7-NR	0.100				
		G	19	10	86.1	24.5-NR	0.400				
	rs3782130	CC	3	2	279.2	12.3-NR	0.400				
		GC	15	7	86.1	24.5-NR					
		GG	30	20	29.4	24.7-NR					
		C	18	9	279.2	24.5-NR	0.200				
		G	45	27	53.6	24.9-NR	0.300				
	rs10877012	TT	3	2	279.2	12.3-NR	0.400				
		GT	15	7	86.1	24.5-NR					
		GG	30	20	29.4	24.7-NR					
		T	18	9	279.2	24.5-NR	0.200				
		G	45	27	53.6	24.9-NR	0.300				
CYP24A1	rs6068816	CC	39	28	28.9	24.5-NR	0.040				
		CT	7	1	NR	NR-NR					
		TT	2	0	NR	NR-NR					
		C	46	29	41.7	24.9-NR	0.200				
		T	9	1	NR	NR-NR	0.010	T	8.492	1.15-62.67	0.0359
	rs4809957	GG	5	2	NR	29.4-NR	0.700				
		GA	15	10	53.6	26.4-NR					
		AA	28	17	45.0	16.8-NR					
		G	20	12	69.9	28.9-NR	0.900				
		A	43	27	53.6	24.7-NR	0.400				
GC	rs7041	TT	12	5	175.2	86.10-NR	0.100				
		TG	22	14	29.6	24.73-NR					
		GG	14	10	23.3	8.67-NR					
		T	34	19	104.3	29.43-NR	**0.060**	T	2.122	0.97-4.66	0.061
		G	36	24	27.7	16.8-NR	0.100				
CYP2R1	rs10741657	GG	23	15	53.6	24.7-NR	0.300				
		GA	23	12	104.3	24.9-NR					
		AA	2	2	20.6	16.8-NR					
		G	46	27	64.5	26.4-NR	0.100				
		A	25	14	86.1	24.5-NR	1.000				
VDR	rs1544410 (BsmI)	AA	7	3	NR	28.9-NR	0.700				
		AG	27	16	29.4	24.5-NR					
		GG	14	10	64.5	21.6-NR					
		A	34	19	41.7	25.6-NR	0.900				
		G	41	26	53.6	24.7-NR	0.400				
	rs11568820 (Cdx-2)	AA	2	2	12.8	8.67-NR	**0.080**				
		AG	16	7	NR	25.60-NR					
		GG	30	20	59.0	24.73-NR					
		A	18	9	29.4	16.8-NR	0.700				
		G	46	27	64.5	26.43-NR	0.040	G	4.345	0.97-19.53	0.0555
	rs2228570 (FokI)	CC	21	12	175.2	26.4-NR	0.400				
		CT	22	13	57.5	13.9-NR					
		TT	5	4	24.9	21.6-NR					
		C	43	25	86.1	25.6-NR	0.300				
		T	27	17	28.9	15.9-NR	0.300				
	rs7975232 (ApaI)	AA	12	7	29.4	21.6-NR	1.000				
		AC	22	12	69.9	24.5-NR					
		CC	14	10	64.5	24.7-NR					
		A	34	19	41.7	24.9-NR	1.000				
		C	36	22	64.5	24.7-NR	0.900				
	rs731236 (TaqI)	CC	6	2	NR	29.8-NR	0.500				
		CT	28	17	29.2	24.5-NR					
		TT	14	10	64.5	21.6-NR					
		C	34	19	41.7	25.6-NR	0.900				
		T	42	27	41.5	24.7-NR	0.300				

MST: median survival time (months)
NR: not reached
Ref Cat: reference category
HR: hazard ratio
IC95%: 95% confidence interval
