# Supplementary material for: Impact of Genetic Polymorphisms on the Metabolic Pathway of Vitamin D and Survival in Non-Small Cell Lung Cancer
Source: Nutrients. 2021 Oct 25;13(11):3783. doi: 10.3390/nu13113783 (PMC8621267; doi:10.3390/nu13113783)
Supplement: Supplementary file 1 [file nutrients-13-03783-s001.zip › Supplementary Files/Table S16.pdf]

Table S16. Polymorphisms and association with progression-free survival of the non-resected NSCLC patients.

Gene	SNPs	Genotype	N	PFS							
				Events	MST (mo)	IC95%	Log-Rank p-value	Ref Cat	Univariate Cox Model		
									HR	IC95%	p-value
CPY27B1	rs4646536	AA	85	84	10.9	8.37-15.5	0.100				
		AG	51	48	10.3	8.50-15.6					
		GG	10	9	20.3	3.77-NR					
		A	136	132	10.9	9.87-13.0	0.040	G	2.05	1.02-4.14	0.0443
		G	61	57	11.2	9.07-17.1	0.600				
	rs3782130	CC	10	9	20.3	3.77-NR	0.100				
		GC	49	46	11.2	8.00-17.1					
		GG	86	85	10.9	8.37-15.5					
		C	59	55	12.0	8.50-17.5	0.500				
		G	135	131	10.9	10.0-13.0	0.040	C	2.05	1.01-4.13	0.0452
	rs10877012	TT	10	9	20.3	3.77-NR	0.100				
		GT	50	47	10.8	8.00-15.6					
		GG	86	85	10.9	8.37-15.5					
		T	60	56	11.6	9.17-17.1	0.500				
		G	136	132	10.9	9.87-13.0	0.040	T	2.05	1.02-4.14	0.0443
CYP24A1	rs6068816	CC	108	103	11.98	10.1-16.1	0.040				
		CT	33	33	10.37	6.20-18.7					
		TT	5	5	5.43	4.27-NR					
		C	141	136	11.17	10.1-14.6	0.010	C	2.99	1.21-7.45	0.0179
		T	38	38	9.68	6.0-14.6	0.400				
	rs4809957	GG	4	4	21.5	4.23-NR	0.900				
		GA	48	46	10.5	8.00-17.1					
		AA	94	91	11.1	9.17-14.6					
		G	52	50	10.5	7.47-17.6	0.700				
		A	142	137	10.9	10.0-13.2	0.700				
GC	rs7041	TT	29	27	10.2	7.57-17.6	0.400				
		TG	67	66	10.5	8.00-15.5					
		GG	50	48	12.4	9.87-17.6					
		T	96	93	10.4	8.37-13.0	0.200				
		G	117	114	11.2	10.0-15.5	0.700				
CYP2R1	rs10741657	GG	52	49	10.3	7.33-16.8	1.000				
		GA	70	69	11.6	10.0-16.1					
		AA	22	21	11.1	7.10-21.8					
		G	122	118	11.1	10.0-14.2	0.900				
		A	92	90	11.2	10.2-16.1	1.000				
VDR	rs1544410 (BsmI)	AA	17	16	9.07	6.53-20.7	0.500				
		AG	75	72	10.70	8.00-14.6					
		GG	54	53	12.83	10.4-17.6					
		A	92	88	10.2	8.0-14.2	0.800				
		G	129	125	11.20	10.2-14.2	0.200				
	rs11568820 (Cdx-2)	AA	9	9	16.13	13.2-NR	0.400				
		AG	53	52	9.17	7.1-11.9					
		GG	84	80	11.70	10.0-16.1					
		A	62	61	10.2	7.47-13.2	0.300				
		G	137	132	10.7	9.07-12.8	0.700				
	rs2228570 (FokI)	CC	64	64	11.3	7.33-15.5	0.400				
		CT	64	59	10.6	10.0-17.6					
		TT	18	18	12.1	10.1-20.7					
		C	128	123	10.7	8.50-14.6	0.600				
		T	82	77	10.9	10.2-17.1	0.300				
	rs7975232 (ApaI)	AA	36	35	9.47	6.70-16.1	0.200				
		AC	71	68	11.20	8.37-16.8					
		CC	39	38	12.90	10.4-17.6					
		A	107	103	10.5	8.37-14.2	0.900				
		C	110	106	11.90	10.3-15.6	**0.060**	C	1.44	0.98-2.13	0.0643
	rs731236 (TaqI)	CC	15	15	7.1	6.20-17.1	0.100				
		CT	75	71	10.7	8.00-16.1					
		TT	56	55	12.8	10.5-17.6					
		C	90	86	10.1	7.70-14.2	0.800				
		T	131	126	11.4	10.3-14.6	0.040	T	1.74	1.01-2.99	0.0463

MST: median survival time (months)
NR: not reached
Ref Cat: reference category
HR: hazard ratio
IC95%: 95% confidence interval
