# Supplementary material for: Impact of Genetic Polymorphisms on the Metabolic Pathway of Vitamin D and Survival in Non-Small Cell Lung Cancer
Source: Nutrients. 2021 Oct 25;13(11):3783. doi: 10.3390/nu13113783 (PMC8621267; doi:10.3390/nu13113783)
Supplement: Supplementary file 1 [file nutrients-13-03783-s001.zip › Supplementary Files/Table S2.pdf]

Table S2. Clinical characteristics and association with progression-free survival of the 194 NSCLC patients.

Characteristic	PFS								
	N	Events	MST (mo)	IC95%	Log-Rank p-value	Reference Category	Univariate Cox Model		
							HR	IC95%	p-value
Gender									
Female	53	43	17.6	12.9-30.0	**0.050**	Female	1.403	0.99-1.99	0.0558
Male	141	127	11.9	9.87-16.1					
Family history									
Yes	102	93	15.6	11.4-19.4	0.200				
No	89	75	12.1	10.2-16.8					
Previous lung disease									
Yes	55	45	17.6	12.7-24.7	0.040	Yes	1.422	1.01-2.01	0.0452
No	139	125	12.3	10.5-16.7					
Smoking status									
Current-Smokers	92	81	13.4	10.30-19.2	0.900				
Former-smokers	75	64	13.2	9.07-18.7					
Non-smokers	27	25	15.6	12.03-20.0					
Alcoholic status									
Current-Drinkers	33	30	9.17	6.7-20.9	0.020	Non-drinkers	1.508	1.00-2.27	0.0485
Former-Drinkers	4	4	7.82	5.1-NR			2.886	1.05-7.95	0.0403
Non-drinkers	124	105	17.07	12.9-20.2			1		
Age at NSCLC diagnosis									
≤60	85	74	11.9	10.2-17.1	1.000				
>60	109	96	15.8	12.8-19.2					
BMI									
<24	31	26	24.5	17.6-51.9	0.200				
>24	83	70	12.9	10.3-18.7					
Histology									
Adenocarcinoma	119	107	14.2	11.4-17.1	0.200				
Squamous cell carcinoma	72	61	12.9	10.0-24.7					
Tumor stage									
I, II and IIIA	63	44	29.4	24.47-86.1	<0.001	I, II and IIIA	3.026	2.11-4.34	<0.001
IIIB and IV	130	125	10.6	9.07-12.8					
First course of treatment									
Surgery	48	29	59.0	25.6-NR	<0.001	Surgery	1		
Chemoradiotherapy	121	118	10.2	7.7-12.9			3.964	2.58-6.08	<0.001
Targeted therapy	25	23	15.8	12.0-21.8			3.233	1.82-5.73	<0.001
First course of treatment (divided by surgery)									
Surgery	48	29	59.0	25.6-NR	<0.001	Surgery	3.838	2.51-5.87	<0.001
No surgery	146	141	10.9	10.0-13.2					

MST: median survival time (months)
NR: not reached
HR: hazard ratio
IC95%: 95% confidence interval
