# Supplementary material for: Impact of Genetic Polymorphisms on the Metabolic Pathway of Vitamin D and Survival in Non-Small Cell Lung Cancer
Source: Nutrients. 2021 Oct 25;13(11):3783. doi: 10.3390/nu13113783 (PMC8621267; doi:10.3390/nu13113783)
Supplement: Supplementary file 1 [file nutrients-13-03783-s001.zip › Supplementary Files/Table S3.pdf]

Table S3. Clinical characteristics and association with overall survival of the resected NSCLC patients.

Characteristic	OS								
	N	Events	MST (mo)	IC95%	Log-Rank p-value	Reference Category	Univariate Cox Model		
							HR	IC95%	p-value
Gender									
Female	13	5	130	114-NR	0.500				
Male	35	17	108	75-NR					
Family history									
Yes	22	14	103	45.9-NR	0.030	No	2.756	1.06-7.20	0.0384
No	23	7	176	107.6-NR					
Previous lung disease									
Yes	22	9	NR	75.0-NR	0.500				
No	23	13	126	88.6-NR					
Smoking status									
Current-Smokers	28	16	114	75-NR	0.300				
Former-smokers	17	5	NR	NR-NR					
Non-smokers	3	1	108	NR-NR					
Alcoholic status									
Current-Drinkers	8	4	75	31.1-NR	0.500				
Non-drinkers	38	18	126	102.5-NR					
Age at NSCLC diagnosis									
≤60	22	9	130	114-NR	0.500				
>60	26	13	108	75-NR					
BMI									
<24	11	3	NR	130.0-NR	0.100				
>24	29	15	114	88.6-NR					
Histology									
Adenocarcinoma	23	9	126	88.6-NR	0.500				
Squamous cell carcinoma	24	13	108	64.7-NR					
Tumor stage									
I, II and IIIA	47	22	130	103-NR	0.600				
IIIB and IV	1	0	NR	NR					

MST: median survival time (months)
NR: not reached
HR: hazard ratio
IC95%: 95% confidence interval
