# Supplementary material for: Impact of Genetic Polymorphisms on the Metabolic Pathway of Vitamin D and Survival in Non-Small Cell Lung Cancer
Source: Nutrients. 2021 Oct 25;13(11):3783. doi: 10.3390/nu13113783 (PMC8621267; doi:10.3390/nu13113783)
Supplement: Supplementary file 1 [file nutrients-13-03783-s001.zip › Supplementary Files/Table S4.pdf]

**Table S4.** Clinical characteristics and association with progression-free survival of the resected NSCLC patients.

| Characteristic | PFS | | | | | | | | |
|---|---|---|---|---|---|---|---|---|---|
| | N | Events | MST (mo) | IC95% | Log-Rank p-value | Reference Category | Univariate Cox Model | | |
| | | | | | | | HR | IC95% | p-value |
| Gender | | | | | | | | | |
| Female | 13 | 6 | 86.1 | 53.6-NR | 0.300 | | | | |
| Male | 35 | 23 | 28.9 | 21.6-NR | | | | | |
| Family history | | | | | | | | | |
| Yes | 22 | 15 | 25.3 | 13.9-NR | 0.100 | | | | |
| No | 23 | 12 | 104.3 | 28.9-NR | | | | | |
| Previous lung disease | | | | | | | | | |
| Yes | 22 | 13 | 67.1 | 25.6-NR | 0.500 | | | | |
| No | 26 | 16 | 59.0 | 13.9-NR | | | | | |
| Smoking status | | | | | | | | | |
| Current-Smokers | 28 | 19 | 27.7 | 16.8-NR | 0.300 | | | | |
| Former-smokers | 17 | 8 | 279.2 | 25.6-NR | | | | | |
| Non-smokers | 3 | 2 | 104.3 | 29.4-NR | | | | | |
| Alcoholic status | | | | | | | | | |
| Current-Drinkers | 8 | 5 | 20.2 | 12.3-NR | 0.600 | | | | |
| Non-drinkers | 38 | 24 | 59.0 | 24.9-NR | | | | | |
| Age at NSCLC diagnosis | | | | | | | | | |
| ≤60 | 22 | 13 | 69.9 | 16.8-NR | 0.800 | | | | |
| >60 | 26 | 16 | 47.1 | 24.7-NR | | | | | |
| BMI | | | | | | | | | |
| <24 | 11 | 8 | 28.9 | 25.6-NR | 0.900 | | | | |
| >24 | 29 | 17 | 86.1 | 24.7-NR | | | | | |
| Histology | | | | | | | | | |
| Adenocarcinoma | 23 | 13 | 86.1 | 16.8-NR | 0.400 | | | | |
| Squamous cell carcinoma | 24 | 16 | 29.4 | 24.7-NR | | | | | |
| Tumor stage | | | | | | | | | |
| I, II and IIIA | 47 | 28 | 64.5 | 25.6-NR | 0.020 | I, II and IIIA | 8.889 | 1.038-76.1 | 0.0461 |
| IIIB and IV | 1 | 1 | 6.9 | NR-NR | | | | | |

MST: median survival time (months)
NR: not reached
HR: hazard ratio
IC95%: 95% confidence interval
