# Supplementary material for: Impact of Genetic Polymorphisms on the Metabolic Pathway of Vitamin D and Survival in Non-Small Cell Lung Cancer
Source: Nutrients. 2021 Oct 25;13(11):3783. doi: 10.3390/nu13113783 (PMC8621267; doi:10.3390/nu13113783)
Supplement: Supplementary file 1 [file nutrients-13-03783-s001.zip › Supplementary Files/Table S5.pdf]

Table S5. Clinical characteristics and association with overall survival of the non-resected NSCLC patients.

Characteristic	OS								
	N	Events	MST (mo)	IC95%	Log-Rank p-value	Reference Category	Univariate Cox Model		
							HR	IC95%	p-value
Gender									
Female	40	31	30.4	23.8-47.4	0.007	Female	1.732	1.15-2.60	0.0082
Male	106	101	20.1	17.1-25.4					
Family history									
Yes	80	74	25.5	16-24.9	0.200				
No	66	58	20.1	21-32.2					
Previous lung disease									
Yes	33	30	21.1	16.0-32.0	0.400				
No	113	102	23.9	19.5-27.9					
Smoking status									
Current-Smokers	64	60	22.1	16.1-27.7	0.600				
Former-smokers	58	53	21.2	17.8-36.5					
Non-smokers	24	19	25.6	23.7-39.6					
Alcoholic status									
Current-Drinkers	25	25	18.43	9.77-26.4	0.002	Non-drinkers	1.95	1.23-3.01	0.0046
Former-Drinkers	4	4	8.95	5.87-NR			3.28	1.12-9.13	0.0229
Non-drinkers	86	73	25.40	22.70-35.2			1		
Age at NSCLC diagnosis									
≤60	63	56	22.1	17.1-30.0	0.400				
>60	83	76	23.9	20.1-30.8					
BMI									
<24	20	17	29.9	22.1-85.5	0.100				
>24	54	48	23.1	18.3-35.2					
Histology									
Adenocarcinoma	96	89	23.4	20.1-26.7	0.100				
Squamous cell carcinoma	48	41	20.6	17.8-41.8					
Tumor stage									
I, II and IIIA	16	11	45.9	32.0-NR	0.004	I, II and IIIA	2.424	1.30-4.51	0.00517
IIIB and IV	129	120	21.1	17.8-24.5					

MST: median survival time (months)
NR: not reached
HR: hazard ratio
IC95%: 95% confidence interval
