# Supplementary material for: Impact of Genetic Polymorphisms on the Metabolic Pathway of Vitamin D and Survival in Non-Small Cell Lung Cancer
Source: Nutrients. 2021 Oct 25;13(11):3783. doi: 10.3390/nu13113783 (PMC8621267; doi:10.3390/nu13113783)
Supplement: Supplementary file 1 [file nutrients-13-03783-s001.zip › Supplementary Files/Table S6.pdf]

Table S6. Clinical characteristics and association with progression-free survival of the non-resected NSCLC patients.

Characteristic	PFS								
	N	Events	MST (mo)	IC95%	Log-Rank p-value	Reference Category	Univariate Cox Model		
							HR	IC95%	p-value
Gender									
Female	40	37	14.2	10.97-19.2	0.050	Female	1.453	0.99-2.12	0.0527
Male	106	104	10.0	7.13-13.0					
Family history									
Yes	80	78	10.8	10.0-15.5	1.000				
No	66	63	11.1	7.70-15.8					
Previous lung disease									
Yes	33	32	10.2	8.0-17.6	0.900				
No	113	109	11.2	10.0-14.6					
Smoking status									
Current-Smokers	64	62	10.6	7.33-14.6	0.900				
Former-smokers	58	56	10.0	7.13-17.1					
Non-smokers	24	23	14.1	11.20-19.2					
Alcoholic status									
Current-Drinkers	25	25	8.37	5.83-20.7	0.090				
Former-Drinkers	4	4	7.82	5.10-NR					
Non-drinkers	86	81	12.48	10.73-17.1					
Age at NSCLC diagnosis									
≤60	63	61	10.4	7.57-12.3	0.900				
>60	83	80	12.8	9.87-16.8					
BMI									
<24	20	18	18.8	16.1-39.1	0.020	<24	1.888	1.09-3.25	0.0218
>24	54	53	10.2	8.0-15.6					
Histology									
Adenocarcinoma	96	94	12.0	10.30-16.1	0.500				
Squamous cell carcinoma	48	45	10.1	7.57-13.0					
Tumor stage									
I, II and IIIA	16	16	17.6	7.70-39.2	0.300				
IIIB and IV	129	124	10.7	9.17-12.8					

MST: median survival time (months)
NR: not reached
HR: hazard ratio
IC95%: 95% confidence interval
