# Supplementary material for: Impact of Genetic Polymorphisms on the Metabolic Pathway of Vitamin D and Survival in Non-Small Cell Lung Cancer
Source: Nutrients. 2021 Oct 25;13(11):3783. doi: 10.3390/nu13113783 (PMC8621267; doi:10.3390/nu13113783)
Supplement: Supplementary file 1 [file nutrients-13-03783-s001.zip › Supplementary Files/Table S7.pdf]

Table S7. Minor allele frequencies of SNPs.

Chr	SNP	Minor Allele	Major Alelle	MAF
4	rs7041	T	G	0.4407
11	rs10741657	A	G	0.3672
12	rs731236	C	T	0.3737
12	rs7975232	A	C	0.4871
12	rs1544410	A	G	0.3866
12	rs2228570	T	C	0.3402
12	rs11568820	A	G	0.2345
12	rs4646536	G	A	0.2448
12	rs3782130	C	G	0.2332
12	rs10877012	T	G	0.2345
20	rs4809957	G	A	0.2088
20	rs6068816	T	C	0.1392
Chr: Chromosome; MAF: Minor allele frequency				
