# Supplementary material for: Impact of Genetic Polymorphisms on the Metabolic Pathway of Vitamin D and Survival in Non-Small Cell Lung Cancer
Source: Nutrients. 2021 Oct 25;13(11):3783. doi: 10.3390/nu13113783 (PMC8621267; doi:10.3390/nu13113783)
Supplement: Supplementary file 1 [file nutrients-13-03783-s001.zip › Supplementary Files/Table S8.pdf]

Table S8. Hardy-Weinberg Equilibrium (OS).

Chr	SNP	Sample	Minor Allele	Major Allele	Genotype counts	Observed heterozygosity	Expected heterozygosity	p-value
4	rs7041	ALL	T	G	41/89/64	0.4588	0.493	0.3817
4	rs7041	AFF	T	G	29/71/54	0.461	0.4868	0.5107
4	rs7041	UNAFF	T	G	12/18/10	0.45	0.4987	0.5371
11	rs10741657	ALL	A	G	24/93/75	0.4844	0.4647	0.642
11	rs10741657	AFF	A	G	21/73/58	0.4803	0.4704	0.8642
11	rs10741657	UNAFF	A	G	3/20/17	0.5	0.4388	0.4894
12	rs731236	ALL	C	T	21/103/70	0.5309	0.4681	0.09064
12	rs731236	AFF	C	T	17/75/62	0.487	0.4573	0.4837
12	rs731236	UNAFF	C	T	4/28/8	0.7	0.495	0.02309
12	rs7975232	ALL	A	C	48/93/53	0.4794	0.4997	0.5677
12	rs7975232	AFF	A	C	39/74/41	0.4805	0.4999	0.6309
12	rs7975232	UNAFF	A	C	9/19/12	0.475	0.4972	0.7582
12	rs1544410	ALL	A	G	24/102/68	0.5258	0.4743	0.1725
12	rs1544410	AFF	A	G	18/76/60	0.4935	0.4628	0.4875
12	rs1544410	UNAFF	A	G	6/26/8	0.65	0.4987	0.1103
12	rs2228570	ALL	T	C	23/86/85	0.4433	0.4489	0.8733
12	rs2228570	AFF	T	C	18/64/72	0.4156	0.4385	0.5809
12	rs2228570	UNAFF	T	C	5/22/13	0.55	0.48	0.513
12	rs11568820	ALL	A	G	11/69/114	0.3557	0.3591	0.8435
12	rs11568820	AFF	A	G	10/53/91	0.3442	0.3617	0.5103
12	rs11568820	UNAFF	A	G	1/16/23	0.4	0.3488	0.6521
12	rs4646536	ALL	G	A	15/65/114	0.3351	0.3698	0.1794
12	rs4646536	AFF	G	A	8/51/95	0.3312	0.3404	0.8122
12	rs4646536	UNAFF	G	A	7/14/19	0.35	0.455	0.1666
12	rs3782130	ALL	C	G	13/64/116	0.3316	0.3576	0.3156
12	rs3782130	AFF	C	G	8/49/96	0.3203	0.3346	0.6283
12	rs3782130	UNAFF	C	G	5/15/20	0.375	0.4297	0.4647
12	rs10877012	ALL	T	G	13/65/116	0.3351	0.3591	0.3243
12	rs10877012	AFF	T	G	8/50/96	0.3247	0.3367	0.6349
12	rs10877012	UNAFF	T	G	5/15/20	0.375	0.4297	0.4647
20	rs4809957	ALL	G	A	9/63/122	0.3247	0.3304	0.828
20	rs4809957	AFF	G	A	5/53/96	0.3442	0.3254	0.6225
20	rs4809957	UNAFF	G	A	04/10/2026	0.25	0.3488	0.07909
20	rs6068816	ALL	T	C	7/40/147	0.2062	0.2396	0.06588
20	rs6068816	AFF	T	C	5/30/119	0.1948	0.226	0.1397
20	rs6068816	UNAFF	T	C	02/10/2028	0.25	0.2888	0.323
Chr: Chromosome								
