# Supplementary material for: Impact of Genetic Polymorphisms on the Metabolic Pathway of Vitamin D and Survival in Non-Small Cell Lung Cancer
Source: Nutrients. 2021 Oct 25;13(11):3783. doi: 10.3390/nu13113783 (PMC8621267; doi:10.3390/nu13113783)
Supplement: Supplementary file 1 [file nutrients-13-03783-s001.zip › Supplementary Files/Table S9.pdf]

Table S9. Hardy-Weinberg Equilibrium (PFS).

Chr	SNP	Sample	Minor Allele	Major Allele	Genotype counts	Observed heterozygosity	Expected heterozygosity	p-value
4	rs7041	ALL	T	G	41/89/64	0.4588	0.493	0.3817
4	rs7041	AFF	T	G	32/80/58	0.4706	0.4883	0.6393
4	rs7041	UNAFF	T	G	09/09/2006	0.375	0.4922	0.2415
11	rs10741657	ALL	A	G	24/93/75	0.4844	0.4647	0.642
11	rs10741657	AFF	A	G	23/81/64	0.4821	0.4702	0.8697
11	rs10741657	UNAFF	A	G	01/12/2011	0.5	0.4132	0.6211
12	rs731236	ALL	C	T	21/103/70	0.5309	0.4681	0.09064
12	rs731236	AFF	C	T	17/88/65	0.5176	0.4601	0.1336
12	rs731236	UNAFF	C	T	4/15/5	0.625	0.4991	0.4115
12	rs7975232	ALL	A	C	48/93/53	0.4794	0.4997	0.5677
12	rs7975232	AFF	A	C	42/80/48	0.4706	0.4994	0.4459
12	rs7975232	UNAFF	A	C	6/13/5	0.5417	0.4991	1
12	rs1544410	ALL	A	G	24/102/68	0.5258	0.4743	0.1725
12	rs1544410	AFF	A	G	19/88/63	0.5176	0.4665	0.1893
12	rs1544410	UNAFF	A	G	5/14/5	0.5833	0.5	0.6826
12	rs2228570	ALL	T	C	23/86/85	0.4433	0.4489	0.8733
12	rs2228570	AFF	T	C	22/72/76	0.4235	0.4496	0.4947
12	rs2228570	UNAFF	T	C	1/14/9	0.5833	0.4444	0.1996
12	rs11568820	ALL	A	G	11/69/114	0.3557	0.3591	0.8435
12	rs11568820	AFF	A	G	11/59/100	0.3471	0.363	0.5316
12	rs11568820	UNAFF	A	G	0/10/14	0.4167	0.3299	0.5394
12	rs4646536	ALL	G	A	15/65/114	0.3351	0.3698	0.1794
12	rs4646536	AFF	G	A	11/56/103	0.3294	0.3536	0.386
12	rs4646536	UNAFF	G	A	04/09/2011	0.375	0.4575	0.3906
12	rs3782130	ALL	C	G	13/64/116	0.3316	0.3576	0.3156
12	rs3782130	AFF	C	G	11/53/105	0.3136	0.3453	0.2632
12	rs3782130	UNAFF	C	G	02/11/2011	0.4583	0.4297	1
12	rs10877012	ALL	T	G	13/65/116	0.3351	0.3591	0.3243
12	rs10877012	AFF	T	G	11/54/105	0.3176	0.3471	0.2708
12	rs10877012	UNAFF	T	G	02/11/2011	0.4583	0.4297	1
20	rs4809957	ALL	G	A	9/63/122	0.3247	0.3304	0.828
20	rs4809957	AFF	G	A	6/56/108	0.3294	0.32	0.8142
20	rs4809957	UNAFF	G	A	03/07/2014	0.2917	0.395	0.2912
20	rs6068816	ALL	T	C	7/40/147	0.2062	0.2396	0.06588
20	rs6068816	AFF	T	C	5/34/131	0.2	0.2253	0.1635
20	rs6068816	UNAFF	T	C	02/06/2016	0.25	0.3299	0.2324

Chr: Chromosome
