# Supplementary figures and images for: Impact of Genetic Polymorphisms on the Metabolic Pathway of Vitamin D and Survival in Non-Small Cell Lung Cancer
Source: Nutrients. 2021 Oct 25;13(11):3783. doi: 10.3390/nu13113783 (PMC8621267; doi:10.3390/nu13113783)

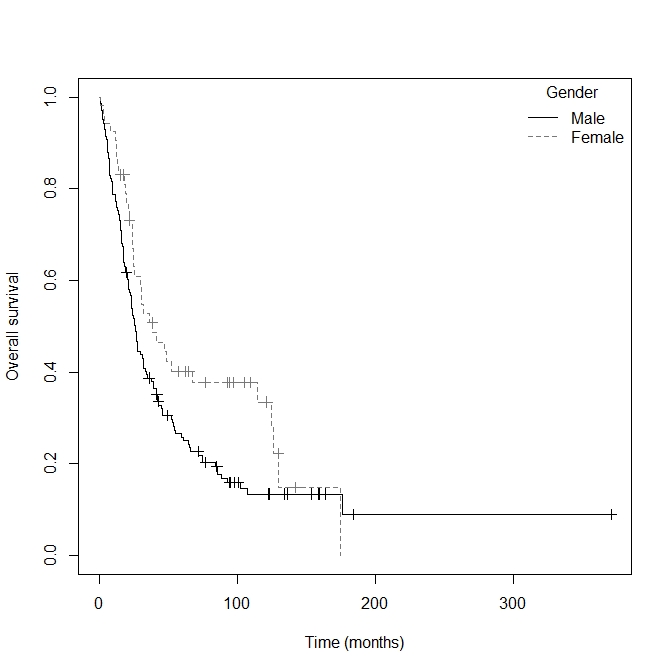

Supplement: Supplementary file 1 [file nutrients-13-03783-s001.zip › Supplementary Files/Figure S1.jpeg]

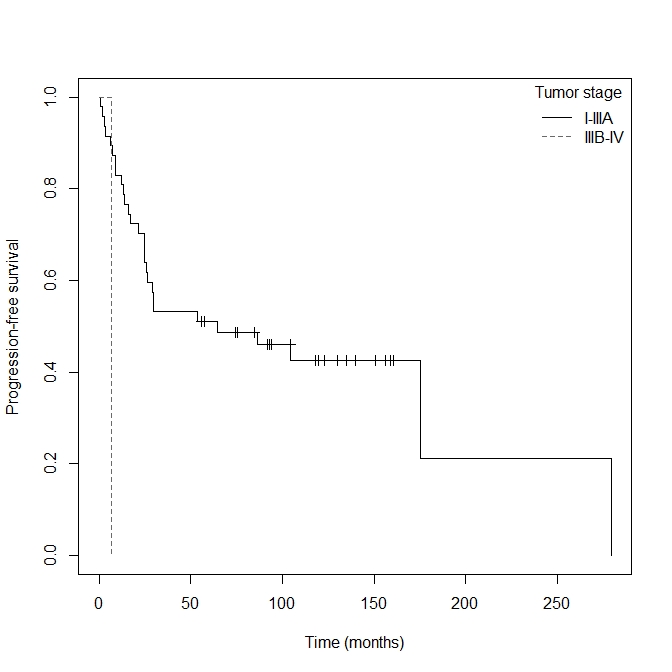

Supplement: Supplementary file 1 [file nutrients-13-03783-s001.zip › Supplementary Files/Figure S10.jpeg]

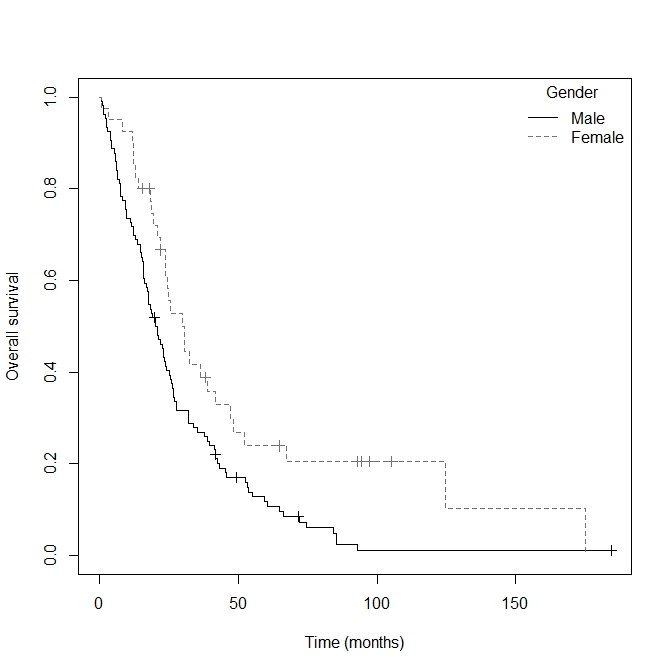

Supplement: Supplementary file 1 [file nutrients-13-03783-s001.zip › Supplementary Files/Figure S11.jpeg]

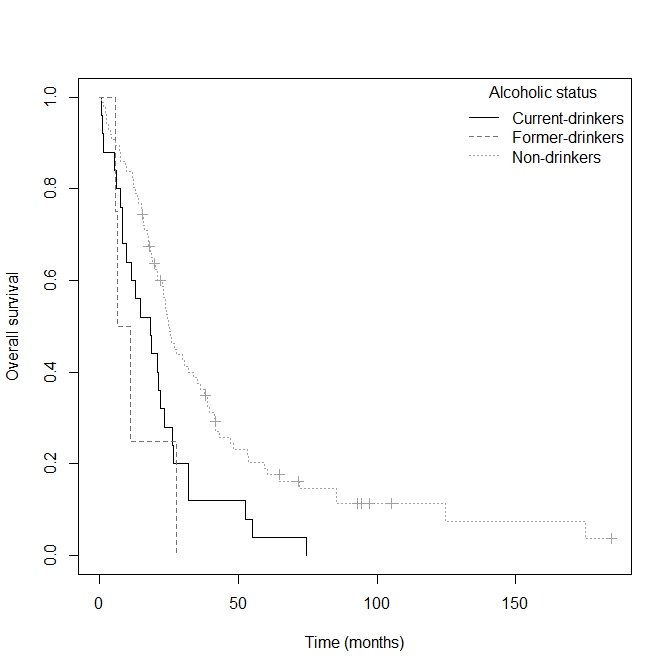

Supplement: Supplementary file 1 [file nutrients-13-03783-s001.zip › Supplementary Files/Figure S12.jpeg]

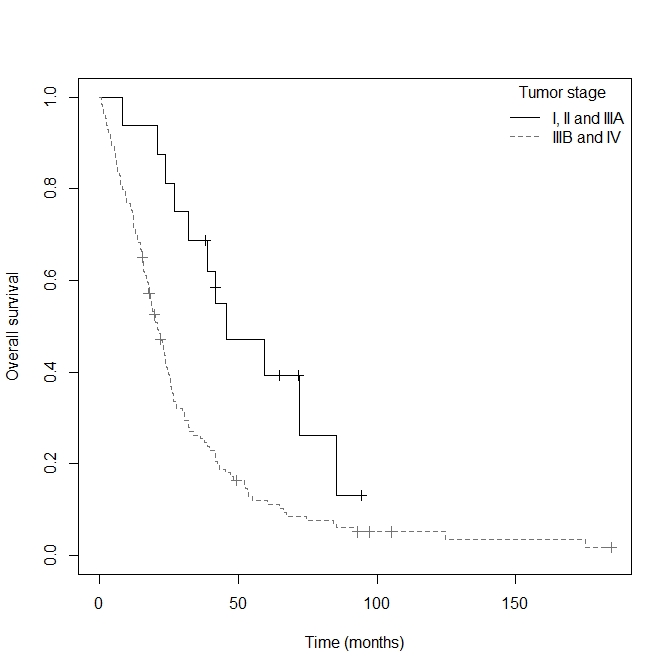

Supplement: Supplementary file 1 [file nutrients-13-03783-s001.zip › Supplementary Files/Figure S13.jpeg]

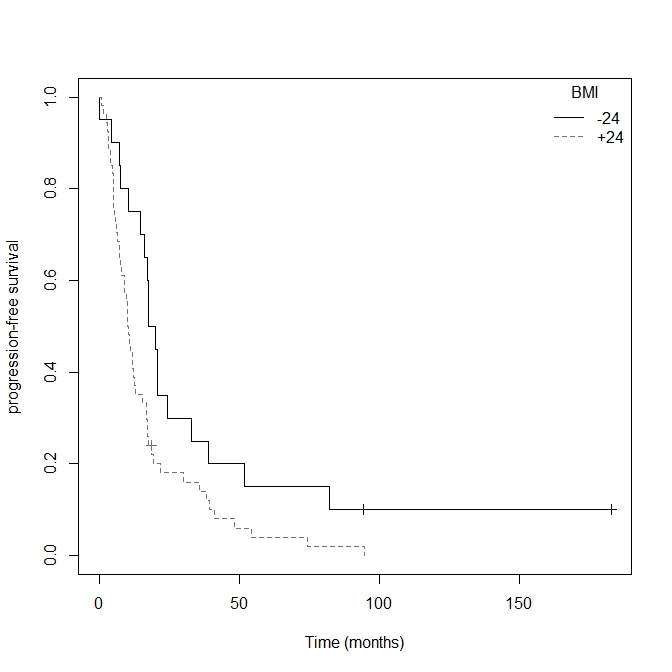

Supplement: Supplementary file 1 [file nutrients-13-03783-s001.zip › Supplementary Files/Figure S14.jpeg]

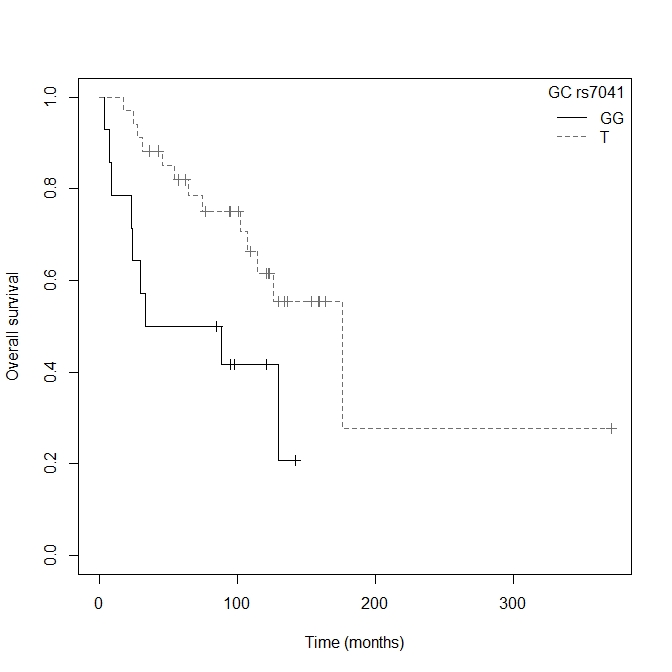

Supplement: Supplementary file 1 [file nutrients-13-03783-s001.zip › Supplementary Files/Figure S15.jpeg]

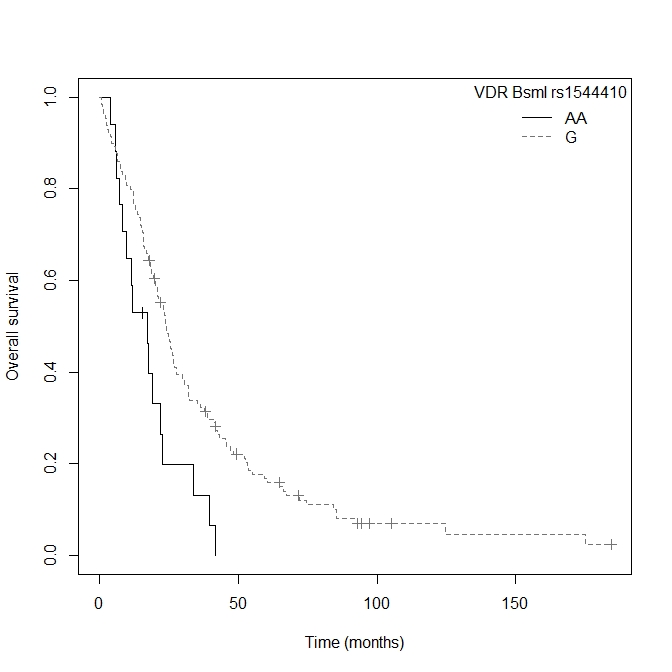

Supplement: Supplementary file 1 [file nutrients-13-03783-s001.zip › Supplementary Files/Figure S16.jpeg]

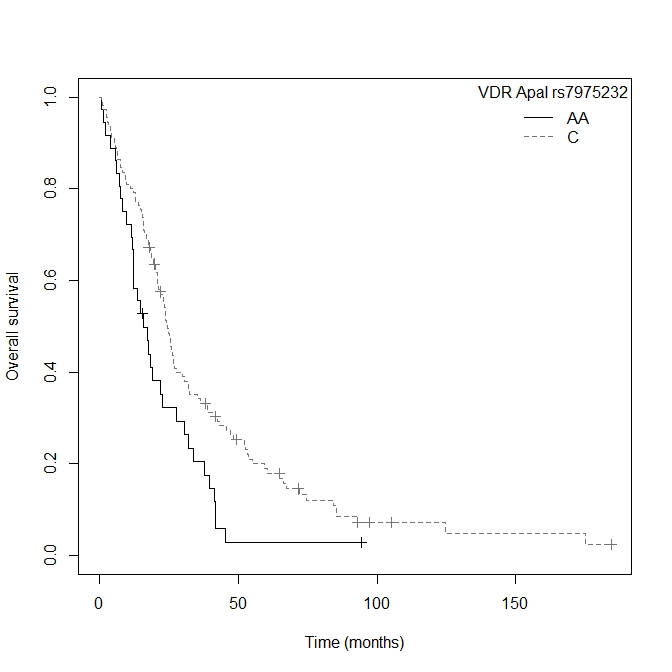

Supplement: Supplementary file 1 [file nutrients-13-03783-s001.zip › Supplementary Files/Figure S17.jpeg]

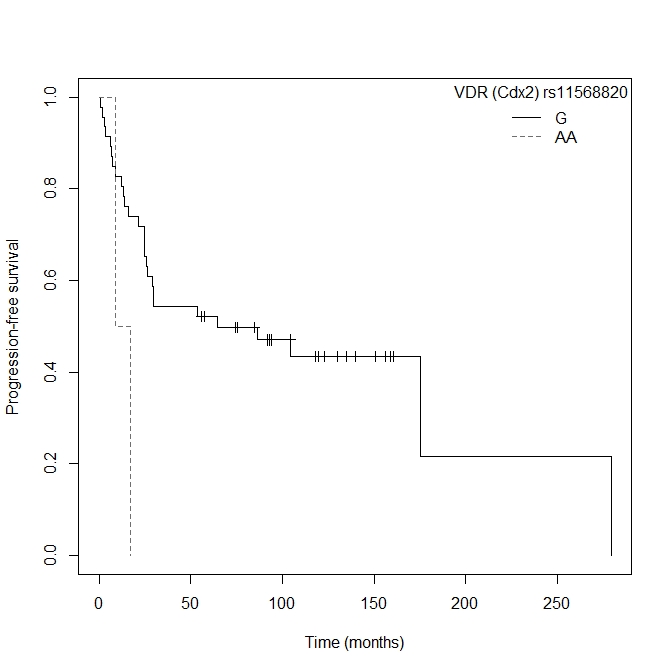

Supplement: Supplementary file 1 [file nutrients-13-03783-s001.zip › Supplementary Files/Figure S18.jpeg]

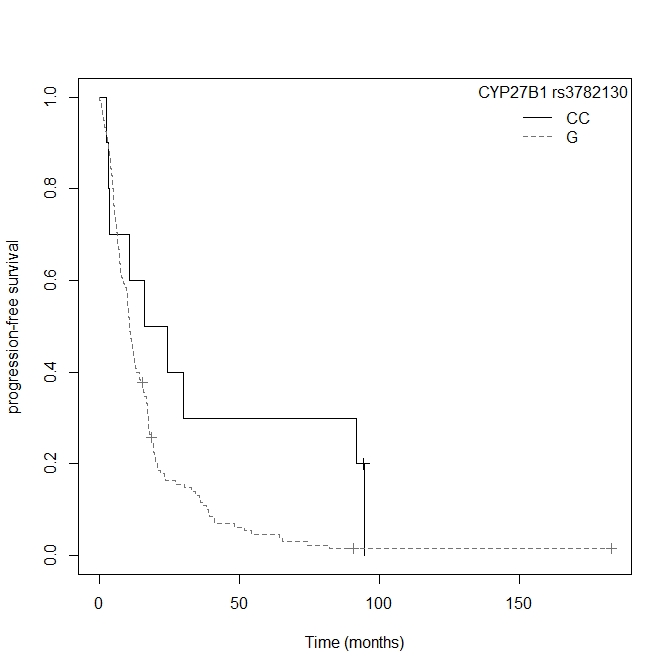

Supplement: Supplementary file 1 [file nutrients-13-03783-s001.zip › Supplementary Files/Figure S19.jpeg]

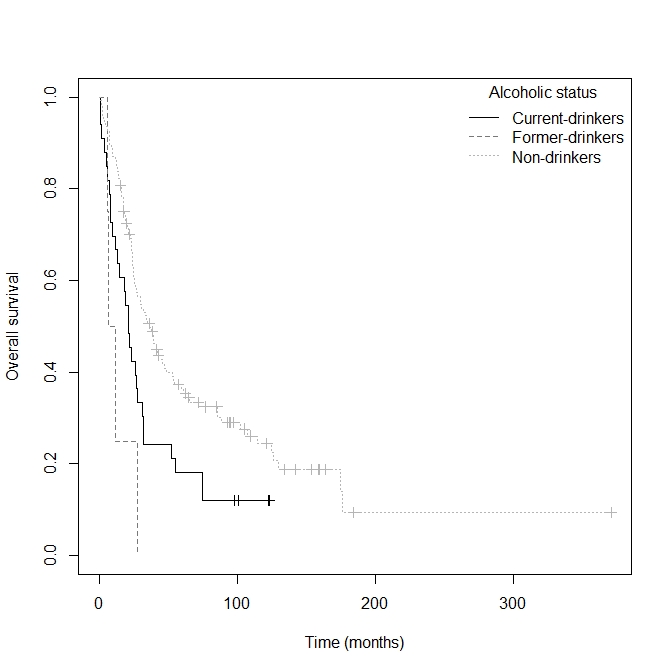

Supplement: Supplementary file 1 [file nutrients-13-03783-s001.zip › Supplementary Files/Figure S2.jpeg]

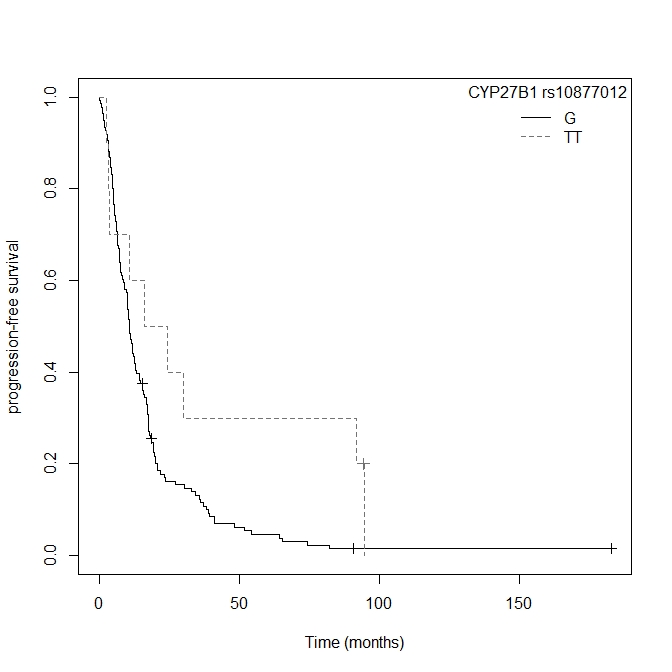

Supplement: Supplementary file 1 [file nutrients-13-03783-s001.zip › Supplementary Files/Figure S20.jpeg]

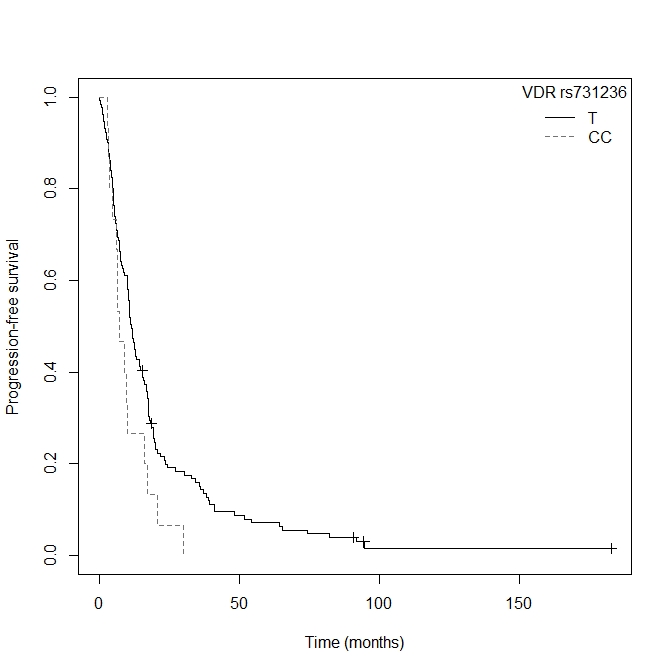

Supplement: Supplementary file 1 [file nutrients-13-03783-s001.zip › Supplementary Files/Figure S21.jpeg]

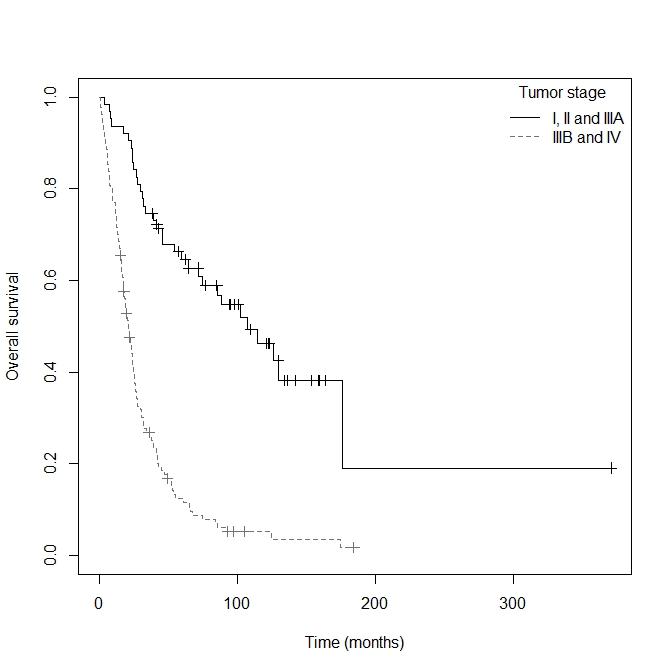

Supplement: Supplementary file 1 [file nutrients-13-03783-s001.zip › Supplementary Files/Figure S3.jpeg]

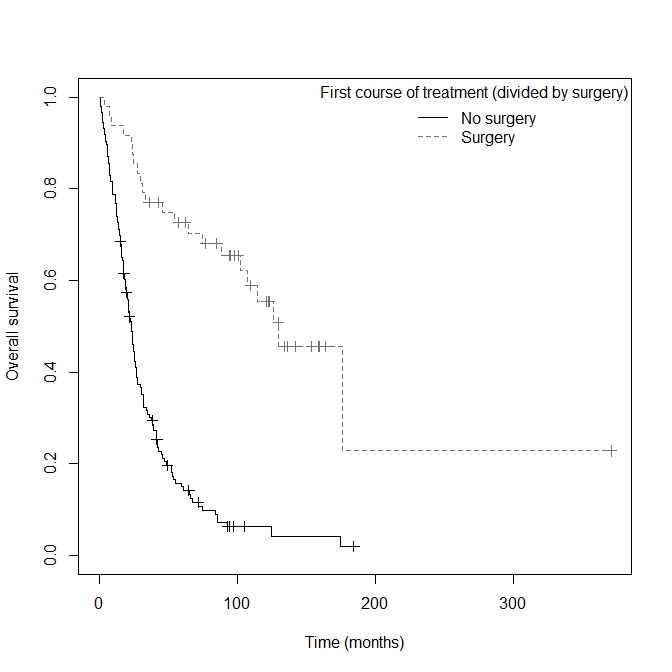

Supplement: Supplementary file 1 [file nutrients-13-03783-s001.zip › Supplementary Files/Figure S4.jpeg]

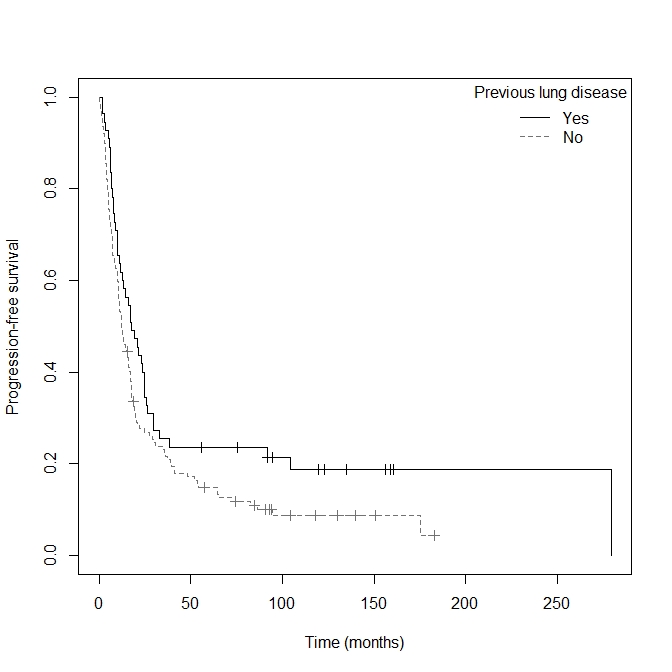

Supplement: Supplementary file 1 [file nutrients-13-03783-s001.zip › Supplementary Files/Figure S5.jpeg]

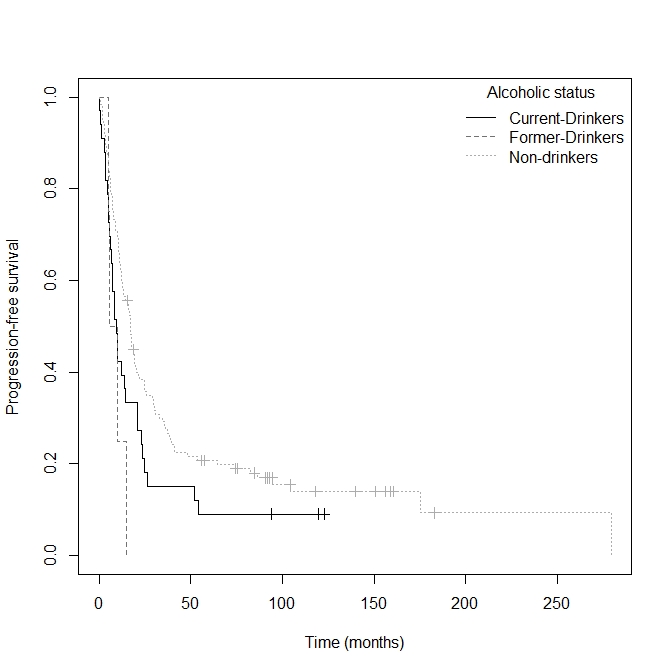

Supplement: Supplementary file 1 [file nutrients-13-03783-s001.zip › Supplementary Files/Figure S6.jpeg]

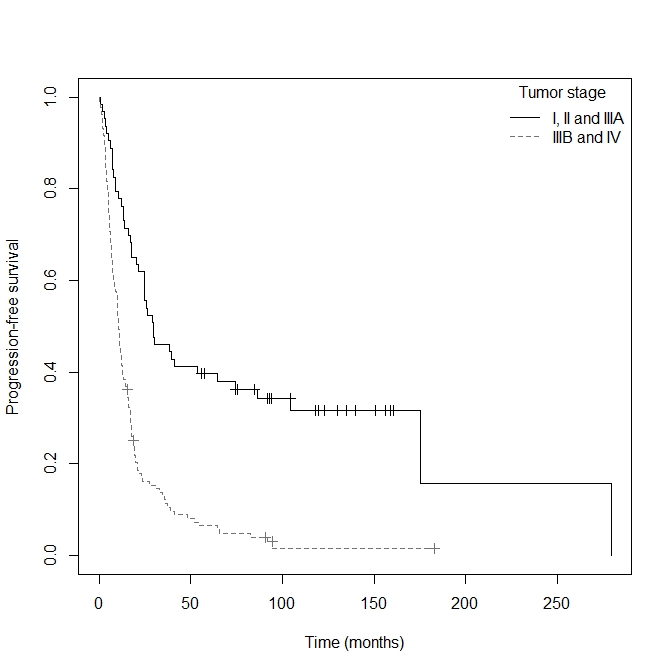

Supplement: Supplementary file 1 [file nutrients-13-03783-s001.zip › Supplementary Files/Figure S7.jpeg]

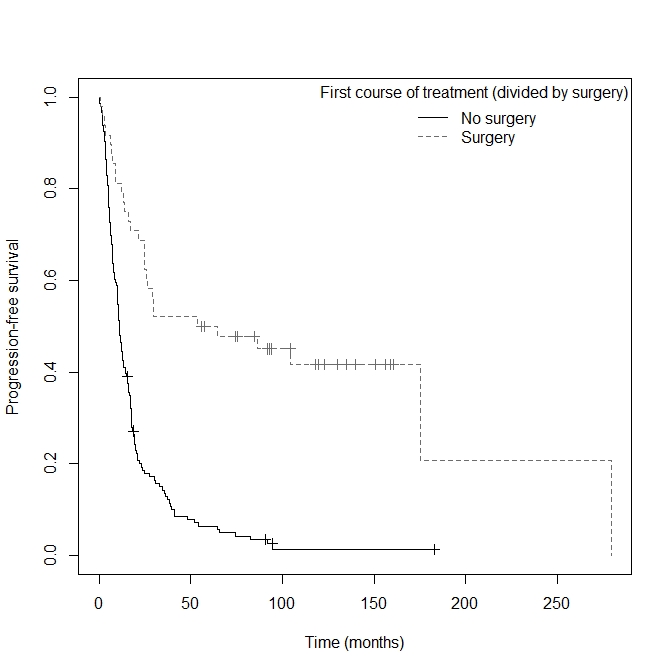

Supplement: Supplementary file 1 [file nutrients-13-03783-s001.zip › Supplementary Files/Figure S8.jpeg]

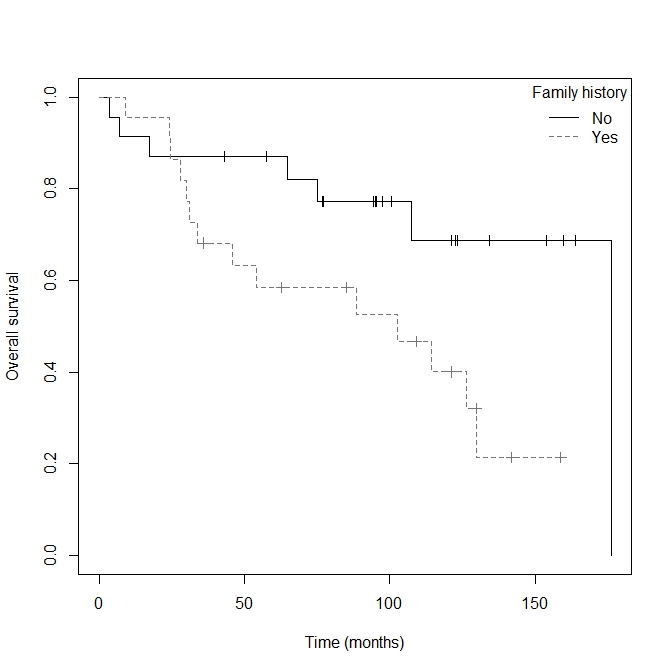

Supplement: Supplementary file 1 [file nutrients-13-03783-s001.zip › Supplementary Files/Figure S9.jpeg]
